# Ribosome profiling reveals changes in translational status of soybean transcripts during immature cotyledon development

**DOI:** 10.1371/journal.pone.0194596

**Published:** 2018-03-23

**Authors:** Md. Shamimuzzaman, Lila Vodkin

**Affiliations:** Department of Crop Sciences, University of Illinois, Urbana, Illinois, United States of America; Universidad Nacional Autonoma de Mexico, MEXICO

## Abstract

To understand translational capacity on a genome-wide scale across three developmental stages of immature soybean seed cotyledons, ribosome profiling was performed in combination with RNA sequencing and cluster analysis. Transcripts representing 216 unique genes demonstrated a higher level of translational activity in at least one stage by exhibiting higher translational efficiencies (TEs) in which there were relatively more ribosome footprint sequence reads mapping to the transcript than were present in the control total RNA sample. The majority of these transcripts were more translationally active at the early stage of seed development and included 12 unique serine or cysteine proteases and 16 2S albumin and low molecular weight cysteine-rich proteins that may serve as substrates for turnover and mobilization early in seed development. It would appear that the serine proteases and 2S albumins play a vital role in the early stages. In contrast, our investigation of profiles of 19 genes encoding high abundance seed storage proteins, such as glycinins, beta-conglycinins, lectin, and Kunitz trypsin inhibitors, showed that they all had similar patterns in which the TE values started at low levels and increased approximately 2 to 6-fold during development. The highest levels of these seed protein transcripts were found at the mid-developmental stage, whereas the highest ribosome footprint levels of only up to 1.6 TE were found at the late developmental stage. These experimental findings suggest that the major seed storage protein coding genes are primarily regulated at the transcriptional level during normal soybean cotyledon development. Finally, our analyses also identified a total of 370 unique gene models that showed very low TE values including over 48 genes encoding ribosomal family proteins and 95 gene models that are related to energy and photosynthetic functions, many of which have homology to the chloroplast genome. Additionally, we showed that genes of the chloroplast were relatively translationally inactive during seed development.

## Introduction

Gene expression is regulated at multiple points such as transcriptional, post-transcriptional, translational, and post-translational levels. Although translation determines the proteome, translational regulation in plants is less well understood compared to other regulatory steps such as transcription and post-transcription. The regulation of gene expression, including at the translational level, is crucial to ensure specific proteins are expressed at the appropriate times and levels in response to genetic and environmental stimuli [1–3]. Thus, the understanding of translational regulation is a major focus in recent years [4–6]. In higher plants, translational regulation plays significant roles in the different developmental processes that control the expression of developmental and stage-specific as well as tissue-specific gene products [7,8].

Genome-wide analyses of gene expression quantify the abundance of mRNA either by microarray or, more recently, by RNA sequencing. However, neither approach provides information on translation of mRNA into protein. Ribosome profiling is a recently developed technique for studying the regulation of gene expression at the translational level [4,9]. This approach is based on high throughput sequencing of ribosome protected mRNA fragments and determines the exact position of ribosomes on mRNA. Generally, transcript abundance is used as the indicator for the gene expression measurement. Sometimes, there is a poor correlation between mRNA abundance and protein levels which is partially due to the translational regulation [6,10]. Whole-proteome mass spectrometry is the direct and powerful approach to measure the changes in protein abundance, but this method can detect only a fraction of protein products in the cell [6]. Ribosome profiling and mass spectrometry are highly complementary approaches to study gene regulation at the translational level. However, ribosome profiling itself allows mRNA abundance and protein translation to be examined in the same sample with high accuracy. One of the advantages of this technique is the measurement of translational efficiency (TE) which is calculated using normalized mRNA abundance and ribosome footprint abundance [4,6]. Higher translational efficiency (TE) values indicate the greater potential of mRNA to be translated into protein.

The maturation phase of soybean seed development has been broadly classified into three major stages. These represent early maturation seed (25–50 mg fresh weight, green seed), mid-maturation (100–200 mg fresh weight, green seed), the stage when the biosynthetic capacity of the seed is maximal and proteins and oils are accumulated at a high rate, and late maturation (300–400 mg fresh weight, yellow seed) when the seed are undergoing dehydration and desiccation [11,12]. Different classes of seed storage proteins, such as glycinin, conglycinin, lectin, and trypsin protease inhibitors, accumulate to high levels during these developmental stages [12–16]. Changes in seed developmental stages are accompanied by changes in gene expression as revealed by transcript profiles of soybean genes [11,12] and their post-transcriptional regulation by small RNAs [17]. So the investigation of changes in the translational status or capacities of transcripts during soybean seed development would add an additional component toward dissecting gene regulatory networks.

Ribosome profiling is an emerging technique which allows us to study the translational potential of all genes during soybean seed development. Sequence information obtained by ribosome profiling can be aligned to the predicted gene models (Glyma models) from the current *Glycine max* reference genome [18], followed by transcript quantification and annotation. TEs calculated from transcripts bound to the ribosomes versus total mRNA transcript levels can be utilized to compare the relative translational values of each gene. Here we used cluster analysis of ribosome profiling data across three developmental stages to determine which genes changed in TE values and thus, their translational capacities, across three stages of soybean seed development.

## Materials and methods

### Plant materials

Soybean (*Glycine max* cv. Williams) plants were grown in a greenhouse and seeds were collected at different developmental stages including early maturation (green 25–50 mg fresh weight seed), mid-maturation (green 100–200 mg fresh weight seed), and late maturation (yellow, 300–400 mg fresh weight seed) as the cotyledons are in the process of desiccation. Immediately, cotyledons and seed coats were separated by dissecting whole seeds and then frozen in liquid nitrogen. Subsequently the tissue was stored at -80°C.

### Preparation of plant cell extracts

A 1 g amount of frozen seed tissue was ground in liquid nitrogen with a mortar and pestle. Multiple beans were used to avoid sampling variation. An aliquot of 100 mg of powdered sample in a microfuge tube was kept frozen at -80°C for total RNA isolation. The remaining powdered sample was homogenized in 10 mL of polysome extraction buffer (PEB) [200mM Tris-acetate pH 8.0, 200 mM sucrose, 200 mM KCl, 10 mM MgCl_2_, 10 mM 2-mercaptoethanol, 2% (v/v) polyoxyethylene (10) tridecyl ether, 1% (v/v) Triton X-100] [3,4,19]. The suspension was filtered through 60 μm nylon mesh and centrifuged at 12,000 rpm for 15 min at 4°C in a JA-17 rotor (Beckman) to remove cell debris. The supernatant was used in the next step for the nuclease digestion.

### Nuclease digestion

At this point, 300 μl of supernatant was transferred to a new tube. Subsequently, 25 μl of CaCl_2_ and 30 μl of micrococcal nuclease (equivalent to 750 units) were added to the supernatant and the reaction mixture was incubated for 1 h at room temperature with gentle rotation of the microfuge tube. In order to stop the digestion reaction, 15 μl (20 U/μl) of SUPERaseIn was added. The samples were kept on ice while preparing for the next steps.

### Purification of ribosome protected mRNA fragments

Ribosome protected mRNA fragments were purified by the Sephacryl S400 spin column (GE Healthcare Life Sciences) chromatography according to the ribosome profiling kit (Epicentre) manuals. This is a rapid and simplified size exclusion spin-column method to isolate monosomes. The Sephacryl S400 columns are usually supplied in a buffer and the resin was re-suspended by inverting the tube several times. Then it was equilibrated by passing through ~300 μl of 1X polysome buffer under gravity flow. Subsequently, 100 μl of nuclease-treated sample was applied onto the column and was centrifuged for 2 minutes at 3000 rpm and the flow through was collected. The ribosome footprint extraction was performed by adding 600–700 μl of RNA extraction buffer and 500 μl of phenol-chloroform. Then the total mixture was centrifuged at 14,000 rpm for 5 minutes at 4°C to separate the phases. The aqueous phase was collected into a fresh 1.5 ml tube and one volume of Sevag (24:1 by volume of chloroform and isoamyl alcohol) was added, shaken vigorously, and centrifuged at 14,000 rpm for 5 minutes at 4°C. After the centrifugation, the aqueous phase was placed in a fresh 1.5 ml tube and 2 μl of glycogen, 1/10th volume of 5 M sodium acetate, and 1.5 volumes of ethanol was added. The reaction mixture was stored at –20°C for at least one hour before centrifugation at 14,000 rpm for 20 minutes to pellet the ribosome footprint fraction. Finally, the ribosome footprint fraction was re-suspended in nuclease-free water and the UV absorbance was taken with the Nanodrop instrument for the next steps of the experiment.

### Control RNA fragment preparation for sequencing

Total RNA was extracted from a 50 mg aliquot of powdered sample using the phenol-chloroform extraction method as described above. The extracted RNA was fragmented using fragmentation buffer (80 mM Tris-OAc, pH ~8.1, 200 mM KOAc, 60mM Mg(OAc)_2_). Then, 20 μl of 2X fragmentation buffer for 20 μl of RNA sample was added. The reaction mixture was incubated for 35 minutes at 95°C. After the incubation, the reaction was stopped by adding 4 μl of 0.5M EDTA.

### Selection of desired fragments

At this point, both the ribosome footprint samples and total RNA samples were analyzed with a 15% TBE (Tris-Borate-EDTA)-urea polyacrylamide gel (Invitrogen). The upper (34 nt) and lower (26 nt) oligonucleotide markers (S1 Table) were used to select the fragments in the range of 26 to 34 nt. Then, the ribosome footprints and RNA fragments were extracted from the gel using the standard protocol [5,6].

### Ribosome footprints and total RNA sequencing library preparation

There were multiple distinct steps for preparation of the sequencing libraries as previously described [4,6,9]. Briefly, Ribo-Zero^™^ rRNA removal kits (Epicentre) were used to reduce rRNA. Both fragmented RNA and ribosome footprint samples were dephosphorylated using T4 polynucleotide kinase (New England Biolabs). After the addition of 1 μl of T4 kinase buffer and 1 μl of T4 polynucleotide kinase, the mixture was incubated for 60 min at 37 °C and then inactivated for 10 min at 70 °C. Dephosphorylated fragments were then purified by precipitation as described above. Preadenylated and 3'-blocked linker (1/5rApp/CTGTAGGCACCATCAAT/3ddC/) (S1 Table) was ligated to the ribosome footprints and RNA fragments. The desired band was excised from the gel and the selected fragments were extracted from the gel.

Subsequently, reverse transcription was performed using the following RT-primer ((Phos)-AGATCGGAAGAGCGTCGTGTAGGGAAAGAGTGTAGATCTCGGTGGTCGC(SpC18)CACTCA(SpC18)TTCAGACGTGTGCTCTTCCGATCTATTGATGGTGCCTACAG) as indicated in [5]. The designation (Phos) represents 5′ phosphorylation, and (SpC18) represents a hexa-ethyleneglycol spacer. After the reverse transcription, the mixture was loaded onto a 15% TBE-urea polyacrylamide gel. The use of the RT-primer as marker facilitated the identification of extended products. These extended products were excised, extracted from the gel, and precipitated as previously described [5,6]. The circularization of gel extracted constructs was performed using CircLigase II (Epicentre). Circularized constructs were used as a template for the final library amplification step using the forward primer (5’-AATGATACGGCGACCACCGAGATCTACAC-3’) and one of the seven indexed reverse primers in S1 Table. Final amplification reactions were performed using 30 s denaturation at 98°C followed by cycles of 10 s denaturation at 98°C, 10 s annealing at 65°C, and 5 s extension at 72°C. Amplification reactions were carried out for 12 cycles as previously described [5,6]. The products were separated on a 10% non-denaturing TBE polyacrylamide gel. The desired band at ~175 nt was cut from the gel and precipitated with sodium acetate and ethanol using standard procedures.

### High-throughput sequencing of libraries and data analysis

High-throughput sequencing for 101 cycles was carried out by the Illumina HiSeq2000 at the Keck Center, University of Illinois at Urbana-Champaign. Sequencing data were filtered through the standard Illumina pipeline including the CASSAVA 1.8.2 program. The 100 nucleotide single sequence reads were trimmed to remove the adapter sequences. Then the trimmed sequences were mapped to all 88,647 high and low confidence soybean gene models from the current assembly Wm82.a2 from Phytozome (Joint Genome Institute) of the Williams 82 genome [18]. These also include all splice variant predictions for 56,044 unique gene models. The ultrafast Bowtie v.1 aligner [20] was used for alignments without any mismatches allowed and with up to 25 alignments to the genome allowed using the following command, v 0 m 25. The most conservative approach is not to allow any mismatches since the short fragment size is predominantly in the range of 26–33 nt. Sequencing reads that aligned to the rRNA sequences of 132 gene models that were falsely annotated by Phytozome as protein containing Glyma models within the nucleolar rDNA region on chromosome 13 were noted. These false annotations result from protein fragments recognized as domains and those reads that matched to the rDNA containing Glyma models were removed from further analysis including determination of the number of mapped reads which yielded 88,515 Glyma cDNA models that were compared. Total hit counts to the Glyma cDNAs from the Bowtie v.1 output were subsequently analyzed by the DESeq package [21] to obtain *p*-values comparing differential expression between the control and footprint samples with two replications. Hit counts of 0 were converted to 1 to avoid losing data in the DESeq analysis. Ribosome profiling and RNA sequencing data were further normalized in reads per kilobase (kb) of gene model per million mapped reads (RPKM) [22] and values were averaged for the two replicates. Alignments to the 152 kb chloroplast genome (NCBI accession DQ317523.1) were conducted with Bowtie v.1 as for alignments to the coding regions of the nuclear genome with no mismatches allowed. RPKMs were calculated in the same way using the number of total reads from each library that were mapped to the 88,515 nuclear gene models as the normalization factors. Trimmed reads from libraries were also aligned to the entire soybean genome Wm82.a2 from Phytozome (Joint Genome Institute) of the Williams 82 genome [18] as before using Bowtie v1 but with output in SAM format. The SAM format was converted to the BAM format and sorted by Samtools [23]. The Integrative Genomic Viewer (IGV) was used to convert from BAM to TDF format and visualize normalized [count at base × one million / total number of reads] data [24]. Raw sequencing data are available in the SRA (short read archive) of the National Center for Biotechnology Information under accession number SRP055880 and series GSE66580 in the GEO database.

### Analysis of translational efficiency (TE)

TE is defined as the number of normalized footprint reads divided by the number of normalized mRNA sequence reads in the form of RPKM [6,9]. TE values were calculated for all the genes across the three seed developmental stages. Gene models were filtered based on their TE values, p-values, and RPKMs as described in the Results section. A higher TE value represents greater potential of mRNA for translation [6,10]. Here we refer to those gene transcripts with TE values >1 as having high values with greater translational potential and those with TE values <0.1 are referred to as having very low TE values and being more translationally silent. The selected genes with high or very low TEs during early, mid and late developmental stages were analyzed by cluster analysis.

### Gene model annotations and clustering

Gene model annotations were obtained from PFAM of the Soybean Genome Project in the Phytozome database [25]. Gene models were grouped into families by manual inspection using these annotations. Clustering of gene models with either high or very low translational values in at least one stage of development was performed by the Multi-Experiment Viewer (MeV) [26]. The k-means clustering technique was used with the Pearson correlation distance metric and 5 x 10^6^ maximum iterations. The correlation coefficients of biological replicates were calculated to show the reproducibility of the ribosome profiling as well as the control RNA sequencing libraries.

## Results

### Developmental stage-specific ribosome profiling libraries, sequencing, and sequence analyses

Ribosome profiling and RNA sequencing libraries were constructed using soybean cotyledons from three different seed developmental stages of early, mid, and late maturation seed. We dissected cotyledons away from the seed coat for these three stages that represent major milestones of seed development such including nutrient accumulation, storage protein synthesis, and desiccation.

**Fig 1:**
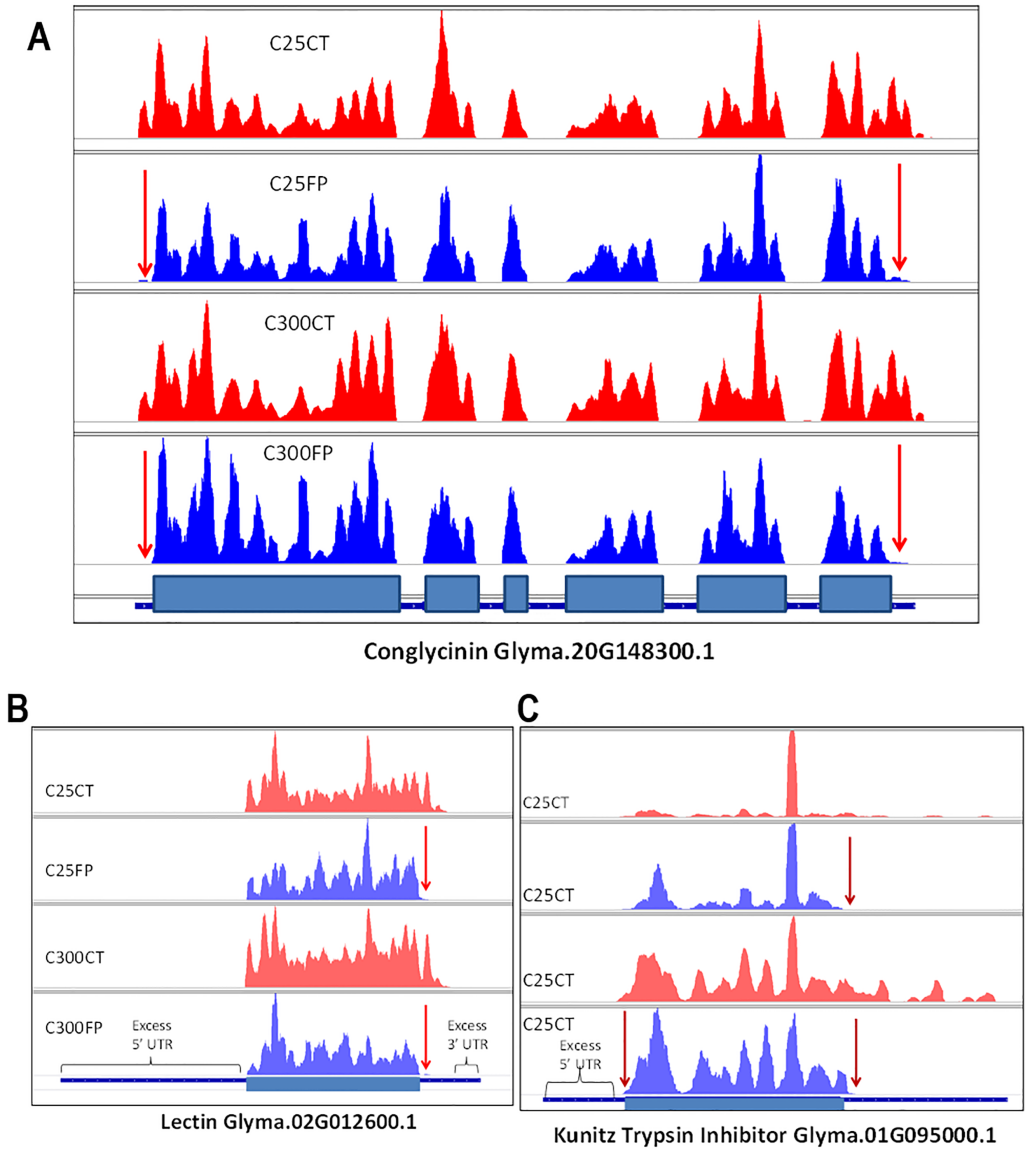
Integrated Genomics Viewer (IGV) displays of base coverage for gene models comparing control (CT) versus ribosome footprint (FP) libraries. Multiple tracks for four libraries show the base coverage versus positions of the indicated gene models from alignments to the soybean reference genome. The C25CT (control) and C25FP (footprint) are libraries of the biological repeat 2 constructed from the 25–50 mg early seed development stage and C300CT and C300FP are from the late stage of 300–400 mg seed weight. The thick blue boxes at the bottom track represent exons and the thin blue boxes are either UTR regions or introns of the indicated gene models for (A) conglycinin, (B) lectin, or (C) Kunitz trypsin inhibitor. Red arrows indicate the absence of coverage in the 5’ and 3’ UTR regions of the footprint libraries compared to the control libraries. Excess 5’ UTR regions in the reference gene models for both lectin and trypsin inhibitor gene models are indicated.

High-throughput next generation sequencing of these libraries was performed on the three stages of soybean seeds, with two biological replicates, resulting in 49 to 113 million reads per sample (S2 Table). These reads were aligned to 88,515 soybean gene models determined by the Soybean Genome Project [18] using the program Bowtie v1 with no mismatches allowed [20]. Read length distributions were found to be very similar between replicates. The read lengths for the control RNA sequencing libraries (S1 Fig) was fairly uniform in the 26–28 nt region and somewhat lower than that of the ribosome footprint libraries which peaked at 31–33 nt (S2 Fig). Data from the two biological replicates are similar as shown in the correlation graph for three stages of cotyledons (S3 Fig). Libraries were reproducible as the Pearson correlation coefficient (r) ranges from 0.8 to 0.97. Data were also aligned to the complete soybean reference genome and displayed with the Integrative Genomics Viewer to assess the quality of the footprint libraries. Fig 1 illustrates the base coverage of selected gene models representing various soybean seed protein genes. The reads aligned to exonic regions only as shown for the conglycinin gene model which has six exons. The footprint libraries also had greatly reduced alignments in the 5’ UTR and 3’ UTR regions as expected. It is also apparent that some gene model calls contain excess 5’ UTR and 3’ UTR regions that are not covered by the control RNA as illustrated for the lectin and trypsin inhibitor Glyma models. In an independent procedure, the numbers of Bowtie v. 1 read alignments to the mRNA transcripts of various gene models were also counted for the 5’ UTR, coding sequence (CDS) and 3’ UTR regions as shown in Table 1. The reduction in alignments for the 5’ and 3’ UTR regions was substantial for each of the seven genes examined indicating that the footprint reads were predominantly located in the CDS regions of the mRNA transcripts examined.

**Table 1:** Sequence reads are predominantly derived from the coding regions of selected gene models for seed storage proteins in the footprint libraries as compared to the control libraries.

Protein Annotation	Gene Model	Library Type	Reads or Normalized Ratio Per Region		
			5' UTR	CDS	3' UTR
Soybean Seed Lectin	Glyma.02G012600.1	C300CT	977	19716	1593
		C300FP	36	10842	22
		FP/CT*	0.07	1.00	0.03
Kunitz Trypsin Inhibitor	Glyma.01G095000.1	C300CT	449	13717	2818
		C300FP	37	7997	64
		FP/CT*	0.14	1.00	0.04
Kunitz Trypsin Inhibitor	Glyma.08G341000.1	C300CT	43	838	139
		C300FP	0	445	4
		FP/CT*	0.00	1.00	0.05
Kunitz Trypsin Inhibitor	Glyma.08G341500.1	C300CT	2159	35460	2559
		C300FP	114	26440	78
		FP/CT*	0.07	1.00	0.04
Conglycinin	Glyma.20G148200.1	C300CT	1564	55629	4953
		C300FP	143	26172	79
		FP/CT*	0.19	1.00	0.03
Conglycinin	Glyma.20G148300.1	C300CT	4495	101409	5075
		C300FP	328	40697	115
		FP/CT*	0.18	1.00	0.06
Conglycinin	Glyma.20G148400.1	C300CT	4453	101409	6464
		C300FP	328	40697	140
		FP/CT*	0.18	1.00	0.05
Glycinin	Glyma.19G164900.1	C300CT	599	22484	1286
		C300FP	10	10100	23
		FP/CT*	0.04	1.00	0.04

The cDNA transcripts derived from the indicated gene models from the soybean genome were used as individual references to which libraries from the C300 stage of 300–400 mg immature seeds were aligned. The specific libraries used were the control library C300CT (RP3001C) compared to its footprint library C300FP (RP3001T). The non-normalized total numbers of trimmed and processed reads aligning to either the 5’ or 3’ UTR regions compared to the protein coding sequences (CDS) of the indicated gene model are shown.

* represents the ratio of the C300FP/C300CT read counts normalized relative to the CDS region as 1.00 and illustrates the dramatic reduction in the representation of the 5’ and 3’ UTR regions in the footprint libraries.

### TE values for the abundant seed storage protein transcripts gradually rise during development after their peak mRNA abundance

**Fig 2:**
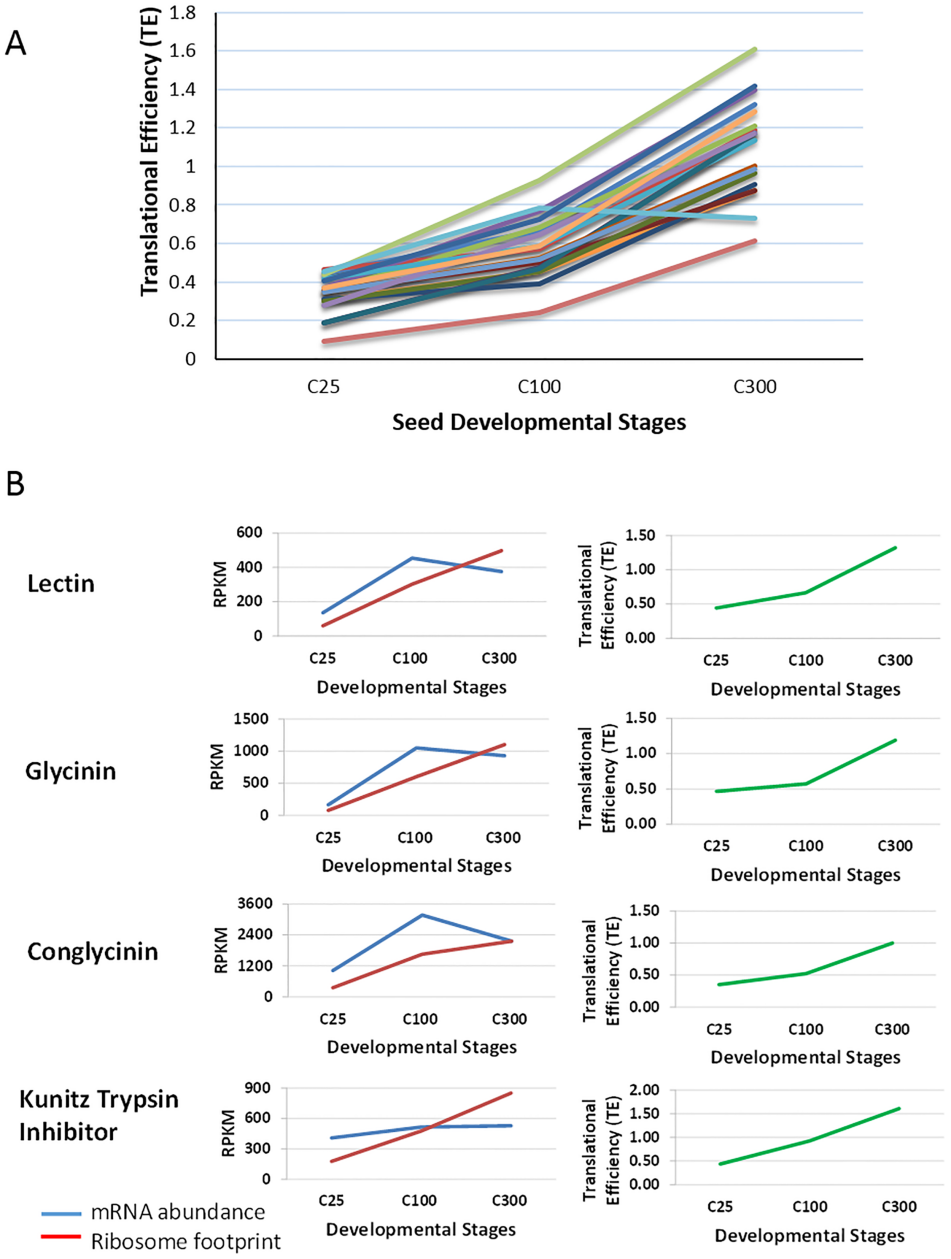
Translational efficiency (TE) and expression profiles of seed storage protein genes during soybean seed development. **(A)** TE values of 19 seed storage proteins at the indicated stages of development from the early stage C25 cotyledons of 25–50 mg, mid stage C100 cotyledons of 100–200 mg, and C300 late stage cotyledons of 300–400 mg. **(B)** Expression profiles (left panel) and TE values (right panel) of representative major seed storage proteins during soybean seed development at the weight ranges indicated. Lectin, Glyma.02G012600.1; Glycinin, Glyma.03G163500.1; Conglycinin, Glyma.20G148300.1; and Kunitz trypsin inhibitor, Glyma.08G341500.1.

Soybean seeds produce several classes of highly abundant storage proteins. The PFAM database annotations for the soybean gene models and all splice variants were filtered by keywords to assemble lists of gene models annotated as in the cupin superfamily and then were verified as soybean seed storage proteins such as glycinin, conglycinin, lectin, and Kunitz trypsin protease inhibitor. These seed storage proteins are the major constituents of soybean seed cotyledons that impart the value of soybean as a high protein crop [27]. We analyzed the expression profiles of these 19 storage protein genes including mRNA abundance, ribosome footprint abundance, and translational efficiency during three stages of seed development (Fig 2 and Table 2). Most of the seed storage protein genes are consistently expressed at much higher levels than most other genes, as expected. Interestingly, whereas seed storage protein transcripts showed their highest level of mRNAs at the mid stage of cotyledon development as expected, their highest ribosome footprint levels were found exclusively at the late stage of seed development. Thus, the proportion of ribosome bound transcripts gradually increases during development for all 19 seed protein genes (Fig 2A and Table 2). A representative gene model from each group of seed storage proteins with their relevant expression profiles are graphed in Fig 2B.

**Table 2:** Major seed storage proteins with normalized (RPKM) expression data for mRNA abundance and ribosome footprint abundance in cotyledons of different seed developmental stages.

Gene Model	C25 CT	C100 CT	C300 CT	C25 FP	C100 FP	C300 FP	C25 TE	C100 TE	C300 TE	Annotation
Glyma.02G012600.1	137	455	377	60	303	498	0.44	0.67	1.32	Lectin
Glyma.03G163500.1	165	1049	928	77	602	1102	0.47	0.57	1.19	Glycinin
Glyma.10G037100.1	85	1082	1277	29	740	1544	0.34	0.68	1.21	Glycinin
Glyma.13G123500.1*	60	1138	1254	22	873	1749	0.37	0.77	1.39	Glycinin
Glyma.19G164900.1	108	702	602	45	413	682	0.42	0.59	1.13	Glycinin
Glyma.02G145700.1	34	135	144	10	62	126	0.30	0.46	0.87	Conglycinin
Glyma.10G028300.1	174	370	359	54	145	325	0.31	0.39	0.91	Conglycinin
Glyma.10G246300.1	839	1896	1311	296	950	1143	0.35	0.50	0.87	Conglycinin
Glyma.10G246500.1	28	78	137	9	35	132	0.30	0.45	0.96	Conglycinin
Glyma.20G146200.1	53	256	1935	10	121	2270	0.19	0.47	1.17	Conglycinin
Glyma.20G148200.1	53	257	1931	10	121	2266	0.19	0.47	1.17	Conglycinin
Glyma.20G148300.1	1016	3173	2157	356	1655	2156	0.35	0.52	1.00	Conglycinin
Glyma.20G148400.1	1015	3165	2162	352	1641	2136	0.35	0.52	0.99	Conglycinin
Glyma.20G146300.1	9	34	186	1	8	114	0.09	0.24	0.61	Conglycinin
Glyma.08G341500.1*	406	515	528	177	477	851	0.44	0.93	1.61	Kunitz trypsin inhibitor
Glyma.01G095000.1	77	438	487	22	282	571	0.28	0.64	1.17	Kunitz trypsin inhibitor
Glyma.08G341000.1	423	252	48	192	198	35	0.45	0.78	0.73	Kunitz trypsin inhibitor
Glyma.08G342300.1	24	28	25	9	17	32	0.37	0.59	1.28	Kunitz trypsin inhibitor
Glyma.09G155500.1	37	14	11	15	10	15	0.41	0.72	1.42	Kunitz trypsin inhibitor

C25 denotes early stage cotyledons, C100 the mid stage, and C300 the late stage of seed development; CT refers to normalized RNA reads; FP refers to normalized ribosomal footprint reads; and TE refers to translational efficiency. Data for biological repeats have been averaged.

* Only the two models indicated by an asterisk passed both the pval <0.05 and TE> = 1 filtering criteria and only in the late stage C300 as shown in the complete data in S1 File.

The seed lectin gene (Glyma.02G012600.1) is the only lectin family member expressed during seed development and results from expression of a single gene [28,29]. Its mRNA abundance sharply increases from 137 RPKMs in the early stage to 455 RPKMs in the mid stage and then it decreases to 377 RPKMs in the late stage (Fig 2B and Table 2). But in the ribosome footprints data, the peak abundance, 498 RPKMs, was found in the late stage. There is not much difference in the translational efficiency (TE) of the seed lectin gene across development, ranging from 0.44 to 1.3. The highest level of relative translational efficiency was found at the late stage which indicates the attachment of more ribosomes to the lectin mRNAs at this stage compared to the other earlier two stages. Glycinin and conglycinin are the two most abundant seed storage proteins and there are multiple members of this family. For a representative glycinin (Glyma.03G163500.1), the peak mRNA abundance was 1049 RPKMs found at the mid stage, whereas a peak ribosome footprint abundance of 1102 RPKMs was found in cotyledons of the late stage. The translational efficiencies of this particular glycinin range from 0.47 to 1.19. Similarly, the representative conglycinin (Glyma.10G246300.1) gene showed the highest mRNA level of 1896 RPKMs at the mid stage, whereas peak footprint abundance was found at the late developmental stage. The translational efficiencies of this conglycinin range from 0.35 to 0.87. We found a similar expression pattern in terms of mRNA abundance, ribosome footprint abundance, and translational efficiencies for other seed storage proteins such as the Kunitz trypsin protease inhibitors. As shown in Table 2, only two transcripts, one for a glycinin family member and another for a Kunitz trypsin inhibitor, passed both our pval <0.05 and TE> = 1 filtering criteria and only in the late stage C300 as shown in the complete data in S1 File.

### Transcripts showing dynamic changes in TE values during development

Both RNA sequencing and ribosome footprint reads were normalized and presented in reads per kilobase of gene model per million mapped reads (RPKMs) [22]. Translational efficiency (TE) was calculated by dividing normalized ribosome footprint reads by the normalized RNA sequencing reads in the form of RPKM [6,10]. TE value is the indicator of how well a particular transcript is translated. Higher TE values indicate more ribosomes are bound to the mRNA and hence there is a higher chance of mRNA translation to synthesize proteins [6,10]. The complete set of ribosome profiling data (S1 File) shows the level of normalized RNA reads, footprint reads and translational efficiency of all soybean annotated genes averaged for both biological replicates and the p-values resulting from DESeq analysis of hit count data from both replicates. Gene models were filtered based on P-values (PVal), translational efficiencies (TEs) and ribosomal footprints (FP) data. Data clustering was also performed using the average of RPKM values from Biological Replicates 1 and 2. In order to obtain transcripts with higher translational potential in different seed developmental stages, all the genes were filtered to retain gene models with Pval<0.05; TE>1 and FP_RPKM> = 1for each of the developmental stages separately. This filter resulted in a final set of 216 unique gene models (excluding alternative splice variants and 13 Glyma models with abnormally high RPKMs), whose transcripts had high TE values in at least one of the developmental stages (Table 3, and the full list in S2 File). We then filtered all the genes with Pval<0.05; TE< = 0.1 and CT_RPKM> = 10 to obtain genes with the more extreme values in which the total mRNA levels were substantially higher than the ribosome footprint values (i.e., very low TE values) during the three stages of development. This resulted in a list of 370 unique gene models with very low TE values that are more translationally silent during at least one of the different developmental stages (Table 3 and S3 File).

**Table 3:** Transcripts that showed changes in TE values during each of three developmental stages.

Category	Total Transcripts			Primary Transcripts			Unique Genes
Developmental Stage	C25	C100	C300	C25	C100	C300	
Transcripts with High TE Values	179	90	53	136	72	46	216
Transcripts with Low TE values	367	237	265	302	218	244	370
CT Transcripts >= 1RPKM	26,317	12,503	13,233	14,888	7,683	8,027	NA

C25 denotes the early stage cotyledons of 25–50 mg seed fresh weight, C100 denotes mid stage cotyledons of 100-200mg, and C300 denotes late stage cotyledons of 300–400 mg. Filtering criteria for high TE values: (Pval<0.05; TE>1 and FP_RPKM> = 1). Filtering criteria for very low TE values: (Pval<0.05; TE< = 0.1 and CT_RPKM> = 10). Pval refers to P-value calculated by the DESeq package, CT refers to normalized control total RNA reads, FP to normalized footprint reads, and TE refers to translational efficiency (TE). The number of transcripts examined was 88,515 total transcripts including all predicted splice variants and 55,912 predicted primary transcripts from the gene models. The unique genes column represents the number of different Glyma models that have either high or very low values by our filtering criteria in at least one of the three developmental stages. NA, not applicable.

### Transcripts with higher translational capacity in at least one stage of cotyledon development

**Fig 3:**
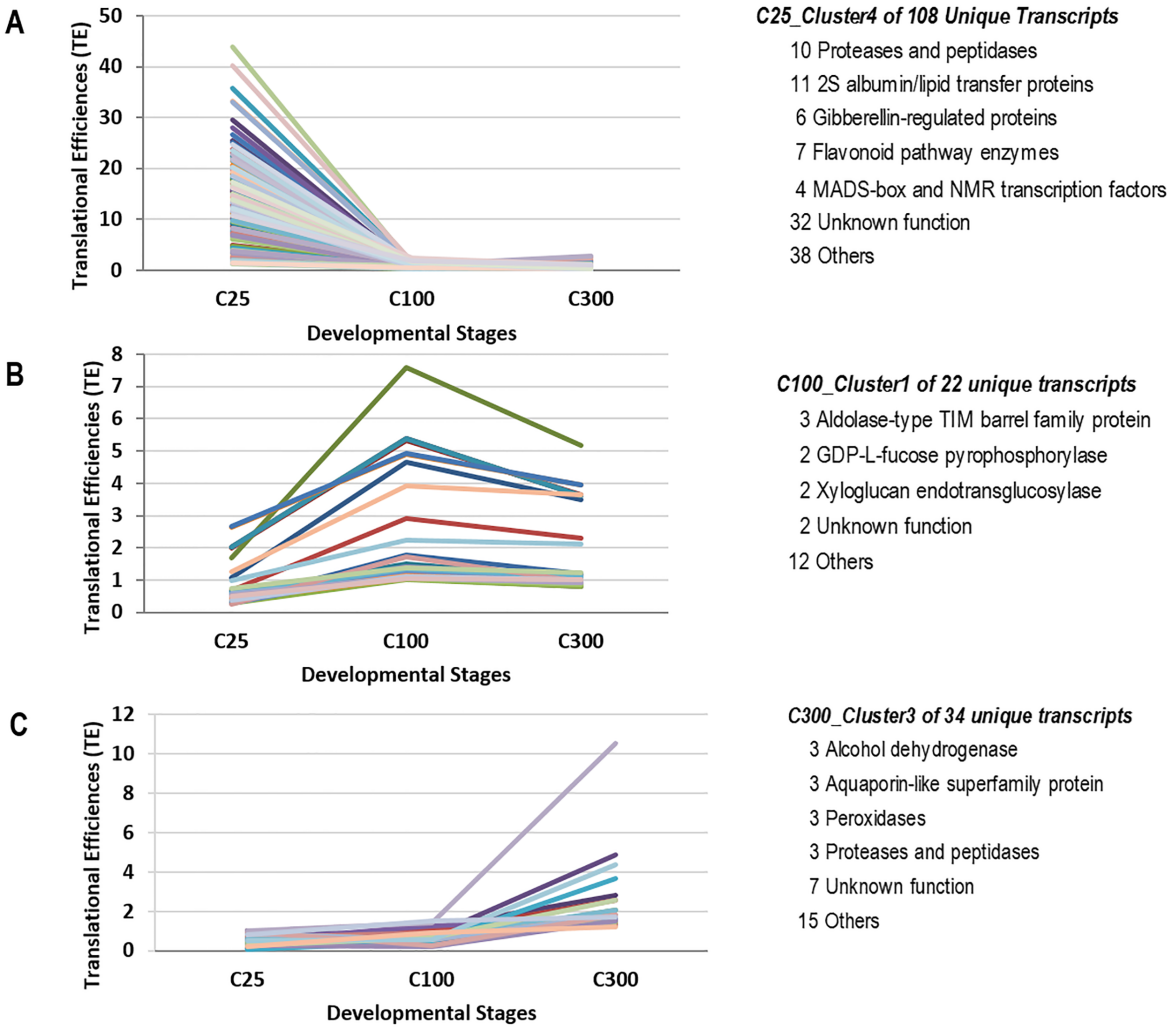
Clusters of transcripts that showed higher translational regulation in at least one stage of seed development. Clusters were created from averaging data from two biological replicates. Full cluster data are shown in S2 File and clustering graphs in S3 File. The left panel displays graphs of TE values of transcripts from representative clusters versus developmental stages for (A) C25 with highest TE values in the early stage (B) C100 with highest TE values in the mid stage and (C) C300 with highest TE values in the late stage. Annotations of the most abundant transcript categories within each cluster are shown in the right panel.

The transcripts with higher TE values shown in the S2 File by stage were then grouped into clusters with the Multi-Experiment Viewer (MeV) [26] as shown in the S3 File. Representative clusters are shown in Fig 3 with the annotations of the most abundant types represented in each cluster.

The representative cluster in Fig 3A contains 108 unique gene models with high TE values ranging from 1.1 to over 43 during the early stage of seed development. In this cluster, ten different gene models, excluding splice variants are annotated, as proteases or peptidases. Other annotation groups in this cluster include a group of proteins with the PFAM annotation “bifunctional inhibitor/lipid-transfer protein/seed storage 2S albumin superfamily protein.” Six transcripts with annotations as gibberellin-regulated proteins and seven various members of the flavonoid pathway stand out as having multiple members along with four transcription factors. The other 37 transcripts were dispersed among many annotation groups each with only one or a few members and another 35 transcripts have unknown functions.

The representative cluster in Fig 3B with 22 unique gene models represents genes that peak in the mid-developmental stage. These transcripts had TE values ranging from 1.02 to over 7.5. Most categories contained only one or at most a few genes. The over-represented annotation groups in this cluster are aldolase-type TIM barrel family protein, GDP-L-fucose pyrophosphorylase, and xyloglucan endo-transglucosylase. Using the same filtering criteria as in the early and mid-stages of development, we identified only 53 transcripts with peak TE values at the late developmental stage that fell into three different clusters. The representative cluster in Fig 3C contains 35 unique gene models with TEs ranging from 1.2 to over 10. Of these, we found the most prominent groups of genes are annotated as alcohol dehydrogenase, aquaporin-like superfamily protein, peroxidases, and eukaryotic aspartyl protease family protein.

**Fig 4:**
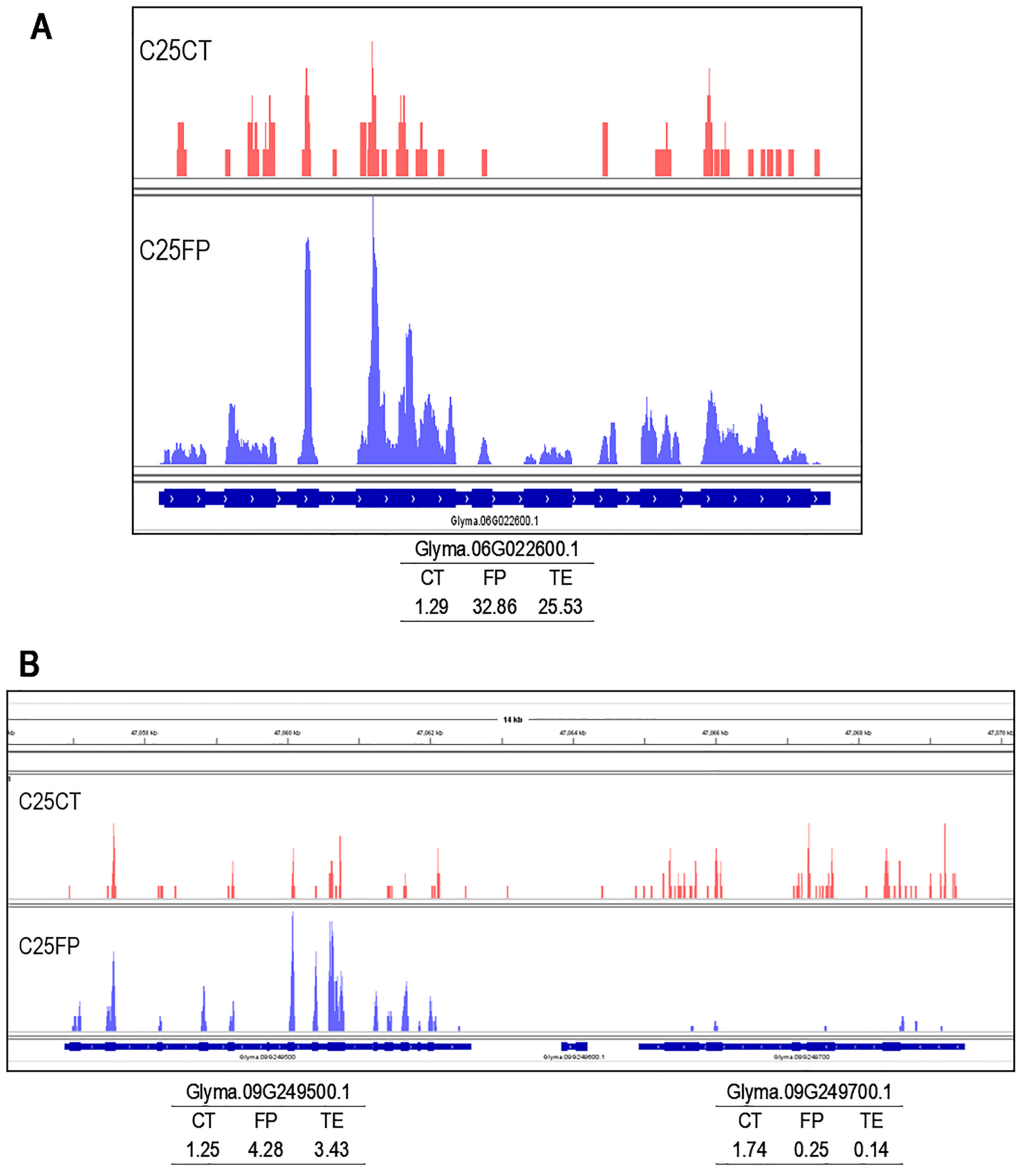
Integrative Genomics Viewer (IGV) displays of base coverage for two serine proteases that exhibit increased TE values. C25CT (control, red) and C25FP (footprint, blue) are libraries constructed from the 25–50 mg early seed development stage. The thick blue boxes at the bottom track represent exons and the thin blue boxes are introns of the indicated gene models. The insets below are the data on RPKMs for the control (CT), footprint (FP), and translational efficiency for the indicated transcripts in (A) Glyma.06G022600 encoding a subtilisin-like serine protease and (B) a 14 kb region with Glyma.09G249500.1 encoding a serine carboxypeptidase as well as a closely linked gene that has a low TE value and encodes a protein phosphatase 2C protein.

Clearly the early C25 stage, and in particular Cluster4, contain the largest number of unique genes that have high TE values. We then searched the entire set of 216 unique transcripts with high TE values in at least one of the three developmental stages (S2 File) for transcripts with abundant annotation classes as protease and peptidases. Table 4 shows that 12 protease and peptidase genes are represented with high TE values in the C25 stage and Fig 4A and 4B show the base coverage for two of these genes, Glyma.06G022600.1 encoding a subtilisin-like serine protease and Glyma.09G249500.1 encoding a serine carboxypeptidase. Both of these have relatively low level expression of approximately 1.3 RPKM in the control RNAs but significantly increased levels of footprint RNAs leading to TE values of 25.5 and 3.4, respectively. A second group of three proteases are represented in the later C300 stage and were more abundantly expressed than those in the early C25 stage.

**Table 4:** Transcripts involved in seed protein storage or mobilization with high TE values in at least one stage of seed development.

Gene Model	C25	C25	C25	C100	C100	C100	C300	C300	C300	Annotation Group^a^
	CT	FP	TE	CT	FP	TE	CT	FP	TE	
Glyma.06G272700.1	0.1	4.02	**40.21**	0.1	0.16	1.58	0.52	0.16	0.31	**A**. cysteine proteinases superfamily
Glyma.02G213000.1	0.1	3.57	**35.67**	0.1	0.1	1.00	0.1	0.1	1.00	**A**. eukaryotic aspartyl protease
Glyma.06G022600.1	1.29	32.86	**25.53**	0.73	0.23	0.32	0.58	0.1	0.17	**A**. subtilisin-like serine endopeptidase
Glyma.09G226700.1	0.16	2.68	**16.4**	0.14	0.1	0.73	0.2	0.1	0.49	**A**. serine carboxypeptidase-like 40
Glyma.04G022500.1	0.21	3.21	**15.11**	0.17	0.1	0.6	0.1	0.1	1.00	**A**. subtilisin-like serine endopeptidase
Glyma.16G018900.1	0.1	1.33	**13.3**	0.1	0.1	1.00	0.1	0.1	1.00	**A**. subtilisin-like serine endopeptidase
Glyma.12G010100.1	0.1	1.25	**12.53**	0.14	0.1	0.7	0.19	0.1	0.53	**A**. serine carboxypeptidase-like 40
Glyma.03G125400.1	0.78	3.04	**3.89**	0.31	0.1	0.32	0.4	0.18	0.45	**A**. serine carboxypeptidase-like 45
Glyma.09G249500.1	1.25	4.28	**3.43**	0.29	0.1	0.35	0.2	0.15	0.74	**A**. serine carboxypeptidase-like 20
Glyma.04G027600.1	1.19	2.91	**2.45**	0.39	0.1	0.26	0.22	0.27	1.23	**A**. papain family cysteine protease
Glyma.18G242900.1	1	1.39	**1.4**	0.31	0.1	0.32	0.16	0.1	0.63	**A**. serine carboxypeptidase-like 20
Glyma.06G027700.1	2.2	2.33	**1.06**	0.53	0.15	0.28	0.27	0.28	1.04	**A**. papain family cysteine protease
Glyma.19G236600.1	0.13	0.1	0.76	13.05	6.82	0.52	130.66	212.14	**1.62**	**B**. eukaryotic aspartyl protease
Glyma.08G116400.1	115.99	52.91	0.46	586.73	671.26	1.14	691.81	1163.3	**1.68**	**B**. xylem bark cysteine peptidase 3
Glyma.03G239700.1	0.43	0.12	0.28	32.14	19.07	0.59	194.63	281.6	**1.45**	**B**. eukaryotic aspartyl protease
Glyma.02G281500.1	0.25	5.02	**20.28**	0.59	0.51	0.86	1.6	0.1	0.06	**C**. LTP/seed storage 2S albumin
Glyma.01G132500.1	1.78	34.05	**19.18**	1.01	0.21	0.21	1.05	0.84	0.79	**C**. lipid transfer protein 1
Glyma.09G058200.1	0.1	1.89	**18.93**	0.1	0.1	1.00	0.1	0.11	1.07	**C**. LMW cysteine-rich 19
Glyma.05G002200.1	0.11	1.92	**17.77**	0.62	0.2	0.32	0.15	0.1	0.66	**C**. LTP/seed storage 2S albumin
Glyma.02G281400.1	0.73	12.64	**17.23**	2.22	0.79	0.35	3.2	1.34	0.42	**C**. LTP/seed storage 2S albumin
Glyma.08G234100.1	0.1	1.64	**16.44**	0.15	0.1	0.68	0.1	0.1	1.00	**C**. LMW cysteine-rich 19
Glyma.19G002300.1	0.23	3.57	**15.82**	0.35	0.19	0.53	0.1	0.17	1.67	**C**. LTP/seed storage 2S albumin
Glyma.03G035700.1	0.21	3.25	**15.32**	0.14	0.1	0.73	0.1	0.1	1.00	**C**. lipid transfer protein 5
Glyma.14G032800.1	0.16	2.16	**13.65**	0.11	0.1	0.94	0.1	0.1	1.01	**C**. LTR/seed storage 2S albumin
Glyma.08G228400.1	0.1	1.2	**11.98**	0.1	0.1	1.00	0.25	0.1	0.39	**C**. LMW cysteine-rich 56
Glyma.20G248700.1	0.18	2.01	**11.19**	0.19	0.34	1.8	0.1	0.59	5.87	**C**. LTP/seed storage 2S albumin
Glyma.10G297600.1	0.23	1.68	**7.37**	1.12	0.71	0.64	0.93	1.51	1.62	**C**. LTP/seed storage 2S albumin
Glyma.09G055100.1	0.25	1.54	**6.07**	0.1	0.35	3.54	0.28	0.49	1.77	**C**. LTP/seed storage 2S albumin
Glyma.09G055000.1	0.34	1.63	**4.83**	0.1	0.38	3.82	0.3	0.53	1.77	**C**. LTP/seed storage 2S albumin
Glyma.05G057800.1	0.97	1	**1.03**	2.75	1.27	0.46	1.96	1.58	0.8	**C**. LTP/seed storage 2S albumin
Glyma.06G067000.1	0.29	0.29	**1**	3.13	3.36	1.07	2.04	2.29	1.12	**C**. LTP/seed storage 2S albumin
Glyma.09G163900.1	0.33	0.2	0.62	1.09	0.63	0.58	1	2.05	**2.05**	**D**. Kunitz family trypsin inhibitor
Glyma.16G211700.1	30.04	11.64	0.39	8.11	8.55	1.05	7.52	7.23	**0.96**	**D**. Kunitz family trypsin inhibitor
Glyma.08G341500.1	406.43	177.39	0.44	514.57	477.37	0.93	528.44	850.64	**1.61**	**D**. Kunitz trypsin inhibitor 1
Glyma.13G123500.1	59.71	21.9	0.37	1138.1	873.26	0.77	1254.2	1748.7	**1.39**	**D**. cupins superfamily/glycinin

RPKMs for control (CT) and footprint (FP) libraries and TE values for the average of the two biological repeats of the indicated stages of development from the early stage C25 cotyledons of 25–50 mg, C100 mid-stage cotyledons of 100–200 mg, and C300 late stage C300 cotyledons of 300–400 mg. Bold type marks the highest TE values for the Glyma model. LTP, lipid transfer protein; LMW, low molecular weight.

^a^Annotation groups: A. Proteases and peptidases with high TE in C25; B. Proteases and peptidases high TE in C300; C. Low molecular weight 2S albumin and other storage proteins with high TE in C25; D. Seed storage proteins with high TE in C300.

Interestingly, there are a total of 16 transcripts with annotations as either 2S albumins/lipid transfer proteins or low molecular weight (LMW) cysteine-rich proteins that also presented high TE values in the early C25 stage. These two classes of proteins have been shown to serve as small molecular weight storage proteins in seeds [27]. One Kunitz trypsin inhibitor and one glycinin are the only two of the 19 major seed storage proteins that met the filtering and p-value criteria as shown in Table 2 previously.

Our data presented in Fig 3 and Table 4 suggest that there may be two phases in the appearance of storage proteins and degradative enzymes in soybean. One is represented by the low molecular weight (LMW) cysteine-rich or 2S albumin proteins and by many serine peptidases and cysteine proteinases in the 25 to 50 mg early maturation seed that have higher TE values. These all have relatively low expression levels. The other phase at the later C300 stage at the 300–400 mg weight range contains some of the more highly expressed proteases but only a few of the major storage proteins. As shown in Fig 2 and Table 2, the abundant storage proteins gradually increase in TE value by approximately 2 to 6-fold from early C25 to the late C300 stages, but most of them do not pass the <0.05 p-value cutoff when comparing the footprint to control RNA-Seq RPKMs, possibly because of the overall very high expression levels of these genes.

Table 5 shows three other major categories found in the transcripts with high TE values including gibberellic acid-regulated genes, flavonoid pathway genes, and four transcription factor genes that are found only in the early C25 stage. They include four gibberellin regulated transcripts and two Glyma models annotated as GAST (gibberellic acid stimulated transcript) protein homolog 1. The occurrence of gibberellic acid is generally biphasic with one peak in early seed development promoting cell growth and expansion and another at a later stage of maturation that induces proteases and germination processes [30]. The flavonoid pathway transcripts include two members for the flavonone 3-hydroxlyases, dihydroflavonol 4-reductases, and leucoanthocyanidin deoxygenases (Table 5). Four transcription factors including two MADS box and two NMR-like negative transcriptional regulators are also represented. These four Glyma models were the only transcription factors present in the complete list of 216 genes with high TE values in at least one stage of development that passed the p-value <0.05 and with FP value >1 RPKM.

**Table 5:** Multiple transcripts of gibberellin-regulated proteins, flavonoid pathway enzymes, and two transcription factors with high TE values in the C25 stage of seed development.

Gene Model	C25	C25	C25	C100	C100	C100	C300	C300	C300	Annotation
	CT	FP	TE	CT	FP	TE	CT	FP	TE	
Glyma.17G258100.1	0.1	2.95	**29.53**	0.24	0.1	0.42	0.1	0.1	1.00	Gibberellin-regulated family
Glyma.13G039300.1	0.3	6.86	**22.69**	0.46	0.28	0.59	0.4	0.1	0.25	Gibberellin-regulated family
Glyma.13G039600.1	0.28	6.16	**21.72**	0.27	0.26	0.97	0.21	0.1	0.47	Gibberellin-regulated family
Glyma.04G024400.1	0.16	2.74	**17.15**	0.18	0.1	0.55	0.1	0.1	0.96	GAST1 protein homolog 1
Glyma.17G258200.1	0.18	2.5	**14.06**	0.1	0.1	1.00	0.24	0.1	0.41	GAST1 protein homolog 1
Glyma.06G044400.1	0.19	1.53	**8.22**	0.1	0.1	0.97	0.2	0.58	2.82	Gibberellin-regulated family
Glyma.02G048400.1	0.34	7.28	**21.41**	0.26	0.1	0.39	0.1	0.1	1.00	flavanone 3-hydroxylase
Glyma.02G048600.1	0.17	2.98	**17.57**	0.14	0.1	0.71	0.1	0.1	1.00	flavanone 3-hydroxylase
Glyma.14G072700.1	0.16	2.46	**15.72**	0.12	0.1	0.83	0.1	0.1	1.00	dihydroflavonol 4-reductase
Glyma.17G252200.4	0.1	1.21	**12.14**	0.1	0.1	1.00	0.1	0.1	1.00	dihydroflavonol 4-reductase
Glyma.05G088100.1	0.1	1.15	**11.5**	0.1	0.1	1.00	0.1	0.1	1.00	flavonol synthase 1
Glyma.11G027700.1	0.2	2.18	**10.74**	0.1	0.1	1.00	0.1	0.1	1.00	leucoanthocyanidin dioxygenase
Glyma.01G214200.1	0.17	1.65	**9.47**	0.1	0.1	1.00	0.1	0.1	1.00	leucoanthocyanidin dioxygenase
Glyma.11G070200.1	0.15	1.74	**11.66**	0.11	0.1	0.92	0.1	0.1	1.00	NmrA-like negative regulator
Glyma.11G070300.1	0.1	1.1	**11.02**	0.16	0.1	0.63	0.1	0.1	1.00	NmrA-like negative regulator
Glyma.06G324400.2	0.22	1.54	**7.08**	0.1	0.1	1.00	0.1	0.16	1.63	MADS-box transcription factor
Glyma.04G257100.2	0.23	1.08	**4.76**	0.1	0.1	1.00	0.1	0.1	1.00	MADS-box transcription factor

RPKMs for control (CT) and footprint (FP) libraries and TE values for the average of the two biological repeats of the indicated stages of development from the early stage C25 cotyledons of 25–50 mg, C100 mid-stage cotyledons of 100–200 mg, and C300 late stage C300 cotyledons of 300–400 mg. GAST, gibberellic acid-stimulated transcript.

### Transcripts with very low TE values in cotyledons

**Fig 5:**
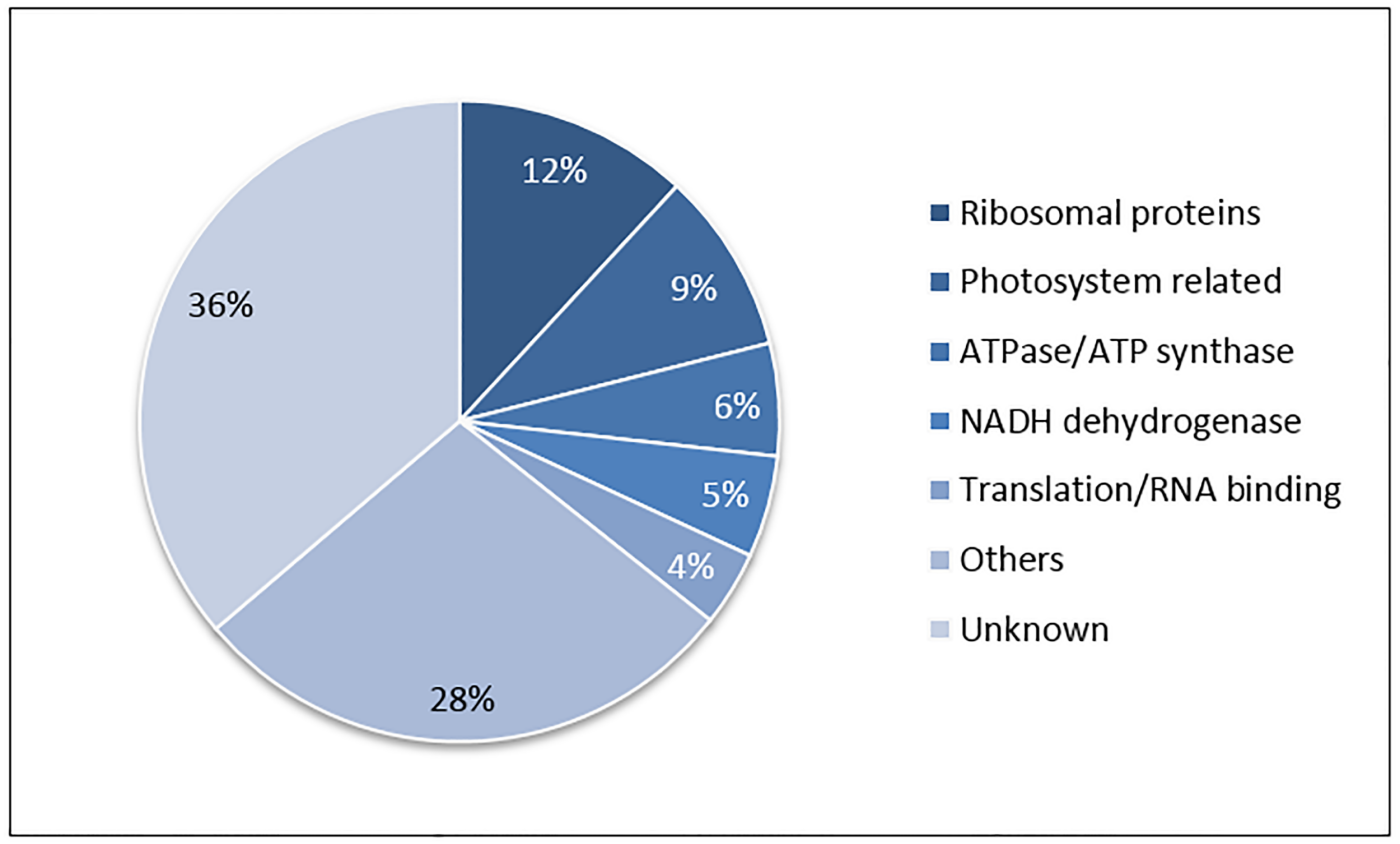
Major annotation groups of 370 transcript that show very low TE values at different seed developmental stages. The 370 unique genes have the following criteria of Pval<0.05; TE< = 0.1 and CT_RPKM> = 10 in at least one of the three developmental stages were grouped into major annotation grouping as percentages.

We then examined the genes with much lower ratios of ribosome bound transcripts versus total mRNA (ie.,very low TE values) by selecting those gene models with Pval<0.05; TE< = 0.1 and CT_RPKM> = 10. We applied the same filtering criteria separately for each developmental stage. We found a total of 302, 218, and 244 primary transcripts in the early, mid and late developmental stages, respectively, (Table 3 and S4 File). These genes of each developmental stage were separately grouped into S5 File with the Multi-Experiment Viewer (MeV) [26]. In contrast to transcript with high TE values, it was clear that the very low TE set of genes shared major annotation groupings in most clusters (S4 File). A representation of the annotation groupings for the entire set of 370 unique transcripts are shown in the pie chart of Fig 5. The largest annotation grouping was clearly the ribosomal proteins at 12% followed by photosynthesis related genes at 9%, including photosystem II reaction center proteins, photosystem I, and PsaA/PsaB proteins, then ATP Synthase/ATPases (6%), NADH dehydrogenases (6%) and translation/RNA binding transcripts at 4%.

These annotation groupings included genes that might be involved in chloroplast and mitochondrial function, as for example the photosystem reaction center proteins, ATPases, the NADH dehydrogenases, and ribosomal proteins. In order to determine Glyma models that have high sequence similarity to the plastid genes, we performed nucleotide Blast alignments of these 370 unique Glyma models against the 152 kb soybean chloroplast genome (NCBI accession DQ317523.1) or the 450 kb soybean mitochondrial genome (NCBI accession JX463295) and flagged the Glyma models that had 90% identity over at least 75% of their coding sequence length (S6 File). Table 6 shows a summary of these results. Of the 48 genes that were annotated as encoding ribosomal proteins, 18 were related to the chloroplast genome and 2 to the mitochondrial genome. The remainder shown in Table 7 represents 28 ribosomal protein genes encoding different structural components of the ribosomes and 14 other Glyma models potentially related to translational processes including several polyA or RNA binding proteins and three family members of the TMA7 (translation machinery associated) proteins. All of these clearly nuclear genes had very low TE ratios indicating that they were relatively silent translationally.

**Table 6:** Ribosomal proteins, translation, and energy related transcripts are overrepresented within the transcripts showing very low TE values.

Match	Annotation Grouping				
	Ribosome	Translation	Energy	Other	Total
Chloroplast	18	0	47	20	85
Mitochondria	2	0	17	10	29
Both	0	0	2	0	2
Neither	28^a^	14^a^	29	183	254
Total	48	14	95	213	370

^a^Table 7 shows the data for these Glyma models.

**Table 7:** Transcripts for 42 ribosomal proteins or proteins involved in translational processes which have very low TE ratios in cotyledons during at least one of the three stages of seed development.

Gene Model	C25 CT	C100CT	C300 CT	C25 FP	C100FP	C300FP	C25 TE	C100TE	C300TE	L	Annotation
Glyma.07G241700.1	18.8	9.5	7.1	1.8	0.8	1.5	0.10	0.09	0.21	186	Ribosomal L29e protein family
Glyma.15G090800.1	16.9	6.9	4.5	1.1	0.8	2.4	0.06	0.12	0.52	210	Ribosomal L38e protein family
Glyma.10G182500.1	20.1	10.2	9.3	2.6	0.9	4.0	0.13	0.09	0.43	399	Ribosomal protein L14
Glyma.19G053400.1	18.6	10.2	13.5	0.6	0.4	0.9	0.03	0.04	0.07	327	ribosomal protein L16
Glyma.03G234600.1	21.0	9.9	8.5	1.6	1.0	2.4	0.08	0.10	0.29	564	ribosomal protein L18
Glyma.20G187300.1	49.6	34.9	82.4	2.4	0.5	3.9	0.05	0.02	0.05	135	ribosomal protein L2
Glyma.11G076000.1	31.1	20.9	11.4	3.6	1.8	3.3	0.12	0.09	0.29	786	Ribosomal protein L2 family
Glyma.08G103700.1	21.8	8.4	5.4	1.7	1.6	2.8	0.08	0.19	0.52	570	Ribosomal protein L22p/L17e family
Glyma.16G169800.1	26.5	10.6	6.7	2.2	2.2	4.0	0.08	0.21	0.60	507	Ribosomal protein L22p/L17e family
Glyma.12G066700.3	30.1	12.4	7.2	5.6	0.8	5.8	0.19	0.06	0.80	375	ribosomal protein L23AB
Glyma.05G223800.1	10.4	5.1	4.6	1.0	0.3	2.7	0.10	0.06	0.59	363	Ribosomal protein L31e family
Glyma.08G030900.1	20.5	12.5	14.5	1.7	0.8	5.4	0.08	0.06	0.37	363	Ribosomal protein L31e family
Glyma.18G119100.1	42.3	29.2	21.6	3.2	2.2	4.7	0.08	0.07	0.22	363	Ribosomal protein L34e superfamily
Glyma.02G253500.1	15.6	8.9	6.2	0.9	0.7	2.1	0.06	0.08	0.34	339	Ribosomal protein L35Ae family
Glyma.14G062900.1	13.0	5.9	5.5	0.7	0.7	1.7	0.05	0.12	0.31	339	Ribosomal protein L35Ae family
Glyma.19G195500.2	62.5	29.1	34.1	4.8	2.5	4.3	0.08	0.09	0.13	345	Ribosomal protein L40e
Glyma.07G088100.2	1.2	26.7	32.6	0.9	1.1	4.8	0.79	0.04	0.15	318	Ribosomal protein S10p/S20e family
Glyma.14G149900.1	10.3	4.7	6.1	0.4	0.1	0.9	0.04	0.02	0.15	267	ribosomal protein S11
Glyma.03G141300.1	22.6	11.5	14.9	1.7	1.4	1.1	0.07	0.12	0.07	216	ribosomal protein S12A
Glyma.09G058100.1	21.5	12.7	14.9	0.4	0.1	1.0	0.02	0.01	0.07	636	ribosomal protein S12A
Glyma.14G213500.1	87.1	56.3	180	3.6	1.5	6.9	0.04	0.03	0.04	141	ribosomal protein S12A
Glyma.10G160300.1	24.8	12.4	9.6	2.6	0.4	2.6	0.10	0.03	0.27	414	Ribosomal protein S24e family
Glyma.20G228100.1	32.7	15.7	10.4	3.2	1.6	4.3	0.10	0.10	0.41	417	Ribosomal protein S24e family
Glyma.16G055600.1	11.0	4.0	3.8	1.0	0.1	2.1	0.09	0.03	0.55	321	Ribosomal protein S26e family
Glyma.02G067400.1	29.0	20.5	17.4	3.5	1.8	6.4	0.12	0.09	0.37	261	ribosomal protein S27
Glyma.13G245200.1	30.3	19.8	23.5	0.2	0.6	0.1	0.01	0.03	0.00	231	Ribosomal protein S7p/S5e family
Glyma.05G173100.1	13.1	6.9	8.5	0.2	0.2	0.2	0.01	0.03	0.02	420	ribosomal protein-related
Glyma.08G104900.1	21.2	25.5	24.3	1.8	2.1	4.0	0.09	0.08	0.17	291	Ribosome membrane protein RAMP4
Glyma.14G221700.1	10.8	4.1	3.1	1.1	0.3	0.8	0.10	0.08	0.25	750	Alba DNA/RNA-binding protein
Glyma.06G197300.2	14.4	11.8	11.8	2.2	0.9	8.1	0.15	0.08	0.68	357	eukaryotic elongation factor 5A-1
Glyma.14G084500.1	22.2	7.9	6.9	1.7	1.1	2.2	0.07	0.14	0.31	1959	poly(A) binding protein 2
Glyma.17G240400.1	26.1	9.1	8.9	2.1	1.0	3.1	0.08	0.11	0.35	1965	poly(A) binding protein 2
Glyma.07G215300.1	37.9	19.8	16.2	3.4	2.6	3.5	0.09	0.13	0.22	1956	poly(A) binding protein 8
Glyma.15G163900.4	20.4	45.9	35.9	0.4	0.5	1.7	0.02	0.01	0.05	2946	pumilio 5
Glyma.07G078300.1	35.9	13.9	20.7	0.6	0.6	1.9	0.02	0.04	0.09	762	RNA-binding (RRM/RBD/RN))
Glyma.02G134400.1	15.7	14.5	14.5	1.4	1.4	3.9	0.09	0.10	0.27	1296	RNA-binding protein 47B
Glyma.07G210800.1	24.8	18.0	17.3	2.3	1.7	4.3	0.09	0.10	0.25	1296	RNA-binding protein 47B
Glyma.07G005600.1	16.3	11.7	11.2	1.0	0.9	2.6	0.06	0.07	0.23	195	Translation machinery TMA7
Glyma.08G206000.1	23.1	15.3	11.9	1.5	0.8	2.3	0.06	0.05	0.19	195	Translation machinery TMA7
Glyma.20G047300.1	18.3	14.9	11.3	1.8	0.8	4.7	0.10	0.05	0.42	195	Translation machinery TMA7
Glyma.13G243100.1	25.6	14.2	19.3	2.5	1.1	2.4	0.10	0.08	0.13	441	Translation protein SH3-like
Glyma.09G044200.1	86.7	83.0	118	5.2	8.2	23.1	0.06	0.10	0.20	507	translationally controlled tumor protein

These 42 Glyma models encode transcripts without substantial identity to the plastid genomes. See S6 File for the full list of all 63 Glyma models in these annotation groups including those with homology to chloroplasts and mitochondria. L, length of the Glyma coding sequence.

Of the 95 genes related to energy/plastid functions, a total of 47 showed significant sequence similarity to the chloroplasts and 17 to the mitochondrial genome and two models had similarity to both organelle genomes (Table 6). Full data for these are shown in S6 File. To determine the profiles of the chloroplast transcripts, we conducted alignments with the original data sets directly against the 152 kb chloroplast genome (NCBI accession DQ317523.1) with no mismatches allowed. As shown in S7 File for all of the coding sequence regions of the chloroplast genome, the highest expression in the control RNA for the CT25 stage was found for the large subunit of the ribulose 1, 5 bisphosphate carboxylase/oxygenase (rbcl) gene with a normalized value of 1411 RPKMs. Many of the photosystem gene transcripts also had relatively high expression levels followed by some of the ribosomal protein genes, then the ATPases, and the NADH oxidoreductases at much lower levels. The normalized RNA footprint levels were consistently lower for all the chloroplast protein genes resulting in very low TE values. All three development stages of the cotyledons exhibited the same general pattern for both the control RNA levels and footprint levels.

## Discussion

There are only a very limited number of studies on ribosome profiling of transcripts on a genome-wide scale in plants, primarily of the model plant *Arabidopsis* [1–3, 31] with very few reports on agronomic crops as maize [32] or legumes [33] and none from soybean or of seed development in any plant. In order to determine the relative translational capacities of transcripts in seed development, we used ribosome profiling experiments of three stages of immature soybean cotyledons. By clustering the TE values into patterns, we found that the TE values varied dynamically for transcripts during seed development. However, the vast majority of soybean genes, including those encoding the major storage proteins, did not demonstrate high TE values during the normal progression of cotyledon development in soybean. The numbers of unique transcripts that were expressed at an arbitrary cut-off level of greater than or equal to 1 RPKM in our control samples were 14,888 in the C25 early maturation 25–50 mg cotyledons, 7,683 in the C100 mid-maturation 100–200 mg cotyledons, and 8,027 in the C300 late maturation 300–400 mg cotyledons (Table 3). Thus, the percentage of transcripts that demonstrated higher ribosome occupancy, ie., TE>1 were 0.91%, 0.94%, and 0.57% in each of the developmental stages, respectively.

To our knowledge there are no reports on translational profiling of immature seed development in any plant species. In *Arabidopsis*, polysomes extracted from the early stages of seed hydration and germination from tissue collected at 0, 6, 26, 48, 72 HAI (hours after imbibition) were profiled on Affymetrix arrays [34]. This approach revealed two shifts in translational regulation, one during the hydration phase in which 435 transcripts had higher TE values and 1204 were low, and another in the germination phase in which 195 transcripts were high and 717 were low.

The immature seed development expression program, especially in a high protein crop as soybean, is dominated by production of large quantities of specific storage proteins. Early molecular studies showed that the abundant storage protein messages account for 50–60% of the polysomal mRNA [35,36]. We found that the number of soybean transcripts showing higher TE values was greatest in the youngest seed at the 25–50 mg stage (Table 3). Examination of our RNA-Seq data [12] from developing Williams cotyledons showed that the number of unique transcripts expressed at > = 1 RPKM was approximately 21,000 to 23,000 in each of several stages of seed development from 5–6 mg, 100–200 mg, and 400–500 mg instead of the 8,000 to 15,000 in this ribosome profiling data. We also observed that the RPKM values of many of the genes were lower by several fold in the control RNA in the ribosome profiling samples. One reason could be that, despite the large sequencing depth of generally over 100 million total sequence reads, more of the sequencing space in the ribosome profiling experiments is occupied by rDNA reads as the technique cannot use poly A selection as used for RNA-Seq, but rather requires fragmentation of the RNA for the control (CT) sample versus the protection provided by the ribosomes in the FP samples. We found only 2–3% of the total mapped sequence reads were to rDNA in the RNA-Seq samples [12] while 20 to 80% of the total mapped reads were aligned to rDNA in the ribosome profiling experiments (S2 Table) and then removed from the analyses informatically. However, the 8.1 to 24.7 million non-rDNA reads mapped to coding transcripts of the Glyma models in the ribosome profiling experiments were sufficient for quantification of most transcripts except possibly for the ones with lowest abundances.

### TE values for serine proteases, 2S albumins, and other LMW storage protein suggest two phases of storage protein mobilization during seed development

The cysteine and subtilisin-like serine proteases [37,38] are the most commonly found seed proteases in soybean and are involved in seed developmental processes including the mobilization and degradation of seed storage proteins in soybean and other legumes [37–43]. Our results showing higher translational efficiencies of serine proteases during early seed development suggest that these proteases might be involved in turning over some proteins before the massive synthesis of the major storage proteins. The expression profiles of the seed storage proteins support this phenomenon where the lowest level of transcripts and footprints is measured in the early stage compared to the mid and late stages (Fig 2 and Table 2). On the other hand, a representative serine protease (Glyma.06G022600.1) has about a 1.3 RPKM level of transcripts in cotyledons of all three stages of seed development, whereas there is a much higher level of footprints, about 32 RPKMs, detected in the early stage compared to the other two stages (Table 4). Hence there is a high level of translational efficiency (TE), at about TE = 25 in the early stage. This implies that there are more ribosomes bound to serine protease mRNAs at this stage which enables more translation. We found eight unique gene models annotated as serine proteases and most of them showed similar expression patterns to the one described above (Table 4). It would appear that the serine proteases play a vital role in the early stages.

In the entire list of 216, there are 16 unique protease gene models that have high TE values in at least one stage of development. Interestingly, several other more abundant proteases, including two aspartyl proteases and a cysteine protease, had higher TE values in the late stage of development after the seeds begin the desiccation process at the 300–400 mg seed weight range (Table 4). Thus, there may be two types of proteases one for the early stages with lower RPKMs but higher TE values, and another class with higher expression and also increased TE values at the more mature stage after the massive increase in storage proteins. These enzymes are likely to play a role in post-germination processes.

The other overrepresented category of transcripts with high TE values possessed the full PFAM annotation of “bifunctional inhibitor/lipid-transfer protein/seed storage 2S albumin superfamily protein”. The 2S albumins and other low molecular weight (LMW) cysteine-rich proteins have been determined to serve as storage proteins in diverse plant species [27,44]. Synthesized initially as preproalbumins, the signal sequence is removed first and the proalbumin intermediates are then proteolytically processed to smaller mature 2S large subunits of 8–10 kd and small subunits of 3–4 kd by endopeptidases and proteases [45]. The mature proteins often also have carboxy-terminal heterogeneity likely from clipping by carboxypeptidases. It has also been shown in some species that both the proteases and peptidases are initially physically separated from each other in multi-vesicular bodies on their way to the storage protein vacuoles [46,47]. Thus, these 16 proteins are likely to be substrates for some of the 12 proteases and peptidases that are present during the early stages of seed development.

It is intriguing that both types of transcripts, those encoding either degradative enzymes or 2S albumins, have generally similar expression and TE patterns in the early C25 stage and then decline in the later stages when a second set of storage proteins and degradative enzymes appears. This suggests two phases to the synthesis and processing and degradation of storage proteins—one represented by the earlier expressed and less abundant class of storage proteins (2S albumins and LMW cysteine-rich proteins) versus the more highly expressed, major globulin proteins represented by glycinin and conglycinin transcripts that accumulate in later stages.

The enhanced translation of the peptidases and 2S albumin proteins perhaps aids in the storage and turnover of amino acids during early seed development and provides a pool of amino acids for the synthesis of the major storage proteins, glycinins and conglycinins. The abundant soybean glycinin and conglycinin proteins and the seed proteins of many important legumes and grains are relatively poor in the essential sulfur amino acids, methionine and cysteine. Although it would be attractive to use some of these 2S albumins as backbones for increasing total sulfur amino acids by engineering, some of them are food allergens as is the S2 albumin of Brazil nuts [44,45] although the 2S albumins in soybean are classified as minor allergens [45].

Whether the six gibberellin-regulated proteins that also have high TE values in the C25 stages are also involved in a network that induces these early 2S albumin proteins and the proteases and peptidases is unknown. However, it has been reported that the pattern of accumulation of gibberellins during seed development shows two peaks corresponding to an early phase when they promote cell growth and expansion and the end of the maturation phase when they activate proteolytic enzymes that are used in germination [30]. The need for higher TE values of the seven flavonoid pathway enzymes at this stage is intriguing. The two copies of similar flavonone 3-hydroxylase gene have previously been cloned and described in detail at the molecular genetic level [48]. One of them was shown to be the *wp* gene that encodes a rare pink flower phenotype in soybean. Flavonoid compounds have many effects on plant development in addition to pigment production. Interestingly, the mutant pink flower line is also associated with a 22% higher seed weight and 4% higher protein content, but the mechanism is unknown.

The molecular mechanisms underlying the observed translational regulation of various mRNAs including alternative expression from upstream orf regions (uORFs) is an attractive subject for further investigation [49,50]. However, one of the current problems with undertaking a complete genomic study of this system would be to first define the UTR for each of the approximately 56,000 unique soybean gene models. For example, as pointed out in Fig 1, the 5’UTR for the seed lectin gene was called as 970 bases, but S1 nuclease protection studies show it to be approximately 28 bp [29]. We have noted the discrepancy in the automated UTR calls made by the Phytozome gene assembly with several of the genes in Table 1 as well. A global experimental determination of the authentic 5’ and 3’ UTR sequences would require correlation of the gene model calls with a number of RNA-Seq data sets from many tissues and organ systems of the soybean plant under various environmental growth conditions including biotic and abiotic stresses.

### Low abundance transcripts encoding MADS box transcription factors and NmrA-like negative regulators showed higher TEs in early development

Translational regulation of mRNAs coding for transcription factors (e. g. yeast GCN4 as the founding example) is a common mechanism of directly connecting cytoplasmic signaling events to altered genome-wide transcriptional program [51,52]. Although transcription factors generally have very low expression, we found two MADS-box transcription factors that had higher TE values in the early stage cotyledons. Members of the MADS-box transcription factor family have a conserved DNA-binding MADS-box domain. The MADS-box family is an important class which is involved in numerous biological processes during plant growth and development [53]. They are mainly recognized to play essential roles in reproductive development [54]. However, MADS-box genes were also found to be expressed in vegetative tissues, root, trichomes, and fruit, suggesting their diverse roles in plant development [55–57]. It has been reported that the soybean MADS-box transcription factors are involved in seed development [58]. We found 9 total members of MADS-box transcription family annotations that demonstrated higher translational efficiency during early seed development, but these contained multiple splice variants of the same two gene models. One MADS-box transcription factor encoding gene, Glyma.06G324400.2, showed translational efficiency of 7.08 in cotyledons during the early stage of seed development, while the other, Glyma.04G257100.2 had a TE of 4.76 (Table 5). The expression of the MADs box is very low in all three stages. However, there is a significant difference in translational efficiencies which indicates more ribosomes are associated with MADS-box transcripts at the early stage. This facilitates the greater potential of MADS-box transcripts to be translated into protein. Our results indicate that MADS-box transcription factors might be playing an important role during the early stage of seed development that requires more protein synthesis than inferred from their transcript levels.

The only other transcription factors with high TE values in the final list of 216 models were two gene models annotated as NmrA-like negative regulators. The wild type NmrA gene is involved in nitrogen metabolite repression in fungi such as *Neurospora crassa* and *Aspergillus nidulans* where it represses the induction of transcription factors that induce genes required to utilize alternative sources of nitrogen other than glutamine or ammonia [59]. In soybean, the principal form of nitrogen delivered to the developing embryo has been measured as glutamine at 55% and asparagine at 20% [60]. Thus, under normal conditions, nitrogen would not be limiting and the possible function of a NmrA-like negative regulator could be envisioned, at least for the early stages of the seed filling period.

### Very low TE values of many ribosomal family proteins and some energy and plastid related transcripts occur across all three developmental stages

Ribosomal proteins are structural constituents of the ribosomes which are necessary for protein synthesis. Interestingly, we found that the footprint values of some ribosomal protein transcripts were much lower than their control levels across all three seed developmental stages, indicating that they are relatively translationally silent during soybean seed development. One such representative ribosomal protein gene (Glyma.14G213500.1) showed very low TE values of 0.04, 0.03 and 0.04 during early, mid and late stages, respectively (Table 7). During the early stage, the transcript level of this particular gene was 87 RPKMs, whereas the footprint level was only 3 RPKMs. Other ribosomal protein coding genes showed similar expression patterns across the developmental stages. All total, 28 of the 370 unique transcripts with very low TE levels were annotated as ribosomal proteins and an additional 14 appeared to be involved in translational machinery (Table 7). At first glance, this result would appear to be at odds with the fact that abundant seed storage protein transcripts need to be associated with functional ribosomes during the mid and late stages of cotyledon development. However, this observation could indicate that there may be specific types of ribosomes that are associated with translation of storage protein transcripts, and thus the levels of some ribosome proteins decrease during seed development to produce that switch. Previously, it has been reported that expression of over 64 ribosomal protein coding genes increased during the transition of the soybean cotyledons from storage organs to photosynthetic organs that occurs at approximately 4 days after seed imbibition and germination [61]. Recently, polysome profiling showed that ribosomes existed primarily as monosomes in *Arabidopsis* dry seed and the polysome fraction increased by six hours after imbibition of the seed [34]. The expression of ribosome protein genes also increased significantly during the seed to seedling transition and the authors speculated that this might affect the composition of the translating ribosomes and the selection of the translated mRNAs [34]. Thus, both transcriptional and translational regulation of ribosome proteins may be important in the immature soybean cotyledons as well as the transition of the cotyledons during seedling growth from a storage organ to a catabolic and subsequently photosynthetic organ. Interestingly, ribosome profiling in embryonic stem cells recently revealed evidence that ribosome heterogeneity at the level of core ribosome proteins facilitates ribosomes to preferentially translate specific mRNAs, mediated in part by internal ribosome entry sites [62]. Thus, in contrast to the current paradigm of ribosomes having conserved structure and function in all tissues, some ribosomes may have structural heterogeneity that leads to functional diversity in their preferences for particular transcripts and subsequent translational preferences. However, many studies would be required to directly verify key compositional differences of ribosomes in the different developmental stages of the seed or other plant parts, as opposed to changes in expression and/or translational capacity of homologous gene family members within each ribosome protein.

Some of the ribosomal proteins (20 of 48 total) and many of the energy, photosynthetic, and plastid related genes (66 of 95 total) have significant homology to the soybean chloroplast and mitochondrial genomes (Table 6). In total, 24% (87 of 370) of the unique Glymas in the very low TE set had identity to the chloroplast genome, whereas only 2% (5 of 216) of the high TE set Glymas had identity to the chloroplast genome (S2 File). The fraction with identity to the mitochondrial genome was lower at 8% (31 of 370) unique Glymas in the very low TE set and none in the higher TE transcripts.

Transfers of DNA have occurred during evolution between the genomes of the nucleus and the organelles. One recent report [63] compared the 450 kb soybean mitochondrial genome from the cultivar Aiganhuang and the 150 kb chloroplast genome [64] from PI 437654 to the assembly of the complete soybean genome from Williams 82 [18]. Their report found the nuclear assembly contained a total of 155.2 kb (0.02%) that had significant identity to the mitochondrial genome with most regions less than 500 bp in length [63]. A larger proportion of the nuclear DNA (1.1 Mb or 0.11%) had significant identities to the chloroplast genome with 24 chloroplast genes having complete ORFs, accounting for 32% of chloroplast coding genes [63].

The large number of nuclear genes or gene fragments with high identity to the chloroplast and mitochondria genomes raises the possibility that our alignments to the Glyma models with these annotations were profiling ribosomes from the organelles, especially since the sequence footprints are necessarily about 30 nt, as that is the size protected by a ribosome from the micrococcal nuclease digestion. Thus, there may be some organelle footprints that align with 100% identity to the nuclear Glyma models. If this is the case, our observations of very low TE values of < = 0.1 for some of the ATPases, NADH dehydrogenases, and photosynthetic reaction center proteins, could be reflecting the relative lack of translation for many of the energy and plastid genes during seed development. Although the early and mid-maturation cotyledons are pale green in color and changes in chloroplast ultrastructure have been observed in the immature cotyledons [65], the developing seed is a sink for photosynthate transported as sugars through the seed coat to the developing embryo. Therefore, the demand for chloroplast functions could be low in the developing cotyledons and/or the pool of RNA in the plastids may be kept high. In order to assess the status of the total RNA versus ribosome bound RNAs that map directly to the chloroplast genome, we aligned our data to the soybean chloroplast genome [64] using the same alignment and normalization criteria that were used for the nuclear coding sequences including no mismatches and using the total reads mapped from each library to the 88,515 nuclear gene models as the normalization factors. As found for the alignments to their nuclear counterparts, the ribosome footprint RNAs of the chloroplast transcripts were highly underrepresented compared to the number of alignments with the control RNA samples as shown in the S7 File representing the 102 coding regions in the chloroplast genome. Thus, it appears that the amounts of unbound polycistronic or processed RNAs in the cotyledon chloroplasts are much higher than those associated with the chloroplast ribosomes. For example, in the C25 stage cotyledons, the chloroplast encoded *rbcl* subunit has the highest level of all the coding sequences at 1411 RPKMs but only 74 RPKMs (0.05 TE value) were found in the footprint sample, and thus associated with active translation of the transcript. The photosystem II protein D2 had the highest TE value at 0.12 (22 FP RPKMs /180 total RPKMs).

A recent study [66] used RNA sequencing to examine the chloroplast translatome in different segments of the 9-day old maize leaf blade. The leaf segments form a natural gradient of cells and plastids at increasing stages of photosynthetic differentiation corresponding to increasing distances from the leaf base. The average TE value for all chloroplast genes in segment 1 was 0.22 and only the *atpH* gene (1.14 average TE) had a TE value >1. The authors found that the translational efficiency of most chloroplast genes increased during development in segments 4 and 9 which are 3–4 cm and 8–9 cm, respectively, from the leaf base, and then declined in segment 14 near the leaf tip as the photosynthetic apparatus matured. In this respect, our results from the cotyledons more closely resemble segment 1 of the leaf base than the other more photosynthetically active tissues of the leaf.

### The major seed storage proteins are primarily regulated at the transcriptional level

During seed development, the accumulation of seed storage proteins is regulated by an integrated genetic and physiological network [67–70]. The glycinins and conglycinins account for up to 70% of the seed protein content with trypsin inhibitors at approximately 5–10% and lectin at 2–5%. The abundant storage protein messages account for 50–60% of the polysomal mRNA [35–36] and lectin and trypsin inhibitor mRNAs were initially enriched and cloned from immunoselection of polysomal RNAs [71]. Lectin and the other seed proteins contain signal sequences that direct their localization to the endoplasmic reticulum and transit to specialized protein body vacuoles in the seed [36,72]. The transcript levels of the abundant storage protein coding genes rise and fall from the early to mid and late maturation phases of seed development as has been shown with several different molecular [13,15,35] and genomics technologies [11–12]. It has already been reported that transcript levels for conglycinin and some other seed proteins are regulated primarily at the transcriptional level during seed development [14,15].

Here, we extensively investigated the expression profiles of the seed storage proteins such as glycinins, conglycinins, lectin, and Kunitz trypsin protease inhibitors in our ribosome profiling data. In contrast to the less abundant 2S albumins, most of the members of these seed storage protein coding genes showed similar expression patterns in cotyledons, where the highest levels of transcripts were found at the mid stage when the seed fresh weight ranges from 100–200 mg (Fig 2 and Table 2). In contrast, the peak ribosome footprint level was found at the late developmental stage even though there was not much difference in relative translational efficiencies across development (Fig 2). These data indicate that although the trend for the seed protein genes is for a gradual increase of 2 to 6-fold in the relative TE values from the early to later stages, these values were only between 1 to 1.6 TE at their highest, indicating roughly equal levels of the total and ribosome bound fractions. Our conclusion is that accumulation of the major storage proteins primarily reflects transcriptional control in line with the massive increase of their transcript levels during seed development. On the other hand, translational regulation may be important to rebalance the proteome to maintain total seed protein levels in situations when naturally occurring mutations or transgenic RNAi knockouts prevent expression of one of the superabundant major storage proteins genes as has been observed [16]. Translational control may also be very important during environmental perturbations as nutrient deprivation or abiotic stress which were not tested in this report.

In summary, in this first report of ribosome profiling of seed development, we profiled both nuclear and chloroplast genomes and identified a relatively small number of genes with high translational efficiencies in cotyledons during different seed developmental stages. Some of them showed higher TE values at only one of the stages of seed development, such as a number of proteases and peptidases and 2S seed storage albumins in the early stage of maturation, suggesting that increased translation of this class of proteins and their degradative enzymes may be needed in the early stage before the major seed proteins increase dramatically. The TE values for the major seed storage protein transcripts increased from 2 to 6 fold TE values from early to late maturation, but their massive accumulation is primarily reflecting the prominence of their transcriptional control during normal seed development. Interestingly, a number of ribosomal protein genes also displayed very low TE values over all three seed developmental stages, even though immature seeds are an extremely active tissue for synthesis of the abundant storage proteins. Genes of the chloroplast appear to be relatively low in translational output during these immature seed stages in which most of the energy is derived from transport of photosynthate into the seed.

## Supporting information

S1 Fig Distribution of RNA sequence reads by length in the biological replicate 1 (left) and biological replicate 2 (right) of cotyledon samples of different seed developmental stages. (TIF)

S2 Fig Distribution of ribosome footprint reads by length in the biological replicate 1 (left) and biological replicate 2 (right) of cotyledon samples of different seed developmental stages. (TIF)

S3 Fig Biological replicates of cotyledon samples show reproducibility within RNA sequencing as well as ribosome profiling libraries. (DOCX)

S1 Table Oligonucleotides used in RNA sequencing and ribosome footprint library preparation. (DOCX)

S2 Table Summary of RNA sequencing and ribosome profiling reads. (DOCX)

S1 File Complete set of normalized ribosome profiling data for all 88,515 gene models and alternative transcripts showing control (CT) RPKMs, footprint (FP) RPKMs, and translational efficiencies (TE) for data from all three developmental stages of immature soybean cotyledons. (XLSX)

S2 File Data from cluster analyses of 322 transcripts (representing 216 different Glyma gene models) selected for high TE values in at least one of the three developmental stages. (XLSX)

S3 File Summary of graphs of all clusters of the gene transcripts with high TE values in at least one of the three developmental stages. (PDF)

S4 File Data from cluster analyses of 764 transcripts (representing 370 different Glyma gene models) selected for very low TE values in at least one of the three developmental stages. (XLSX)

S5 File Summary of graphs of clusters of the transcripts with very low TE values in at least one of the three developmental stages. (PDF)

S6 File Annotation categories of energy, ribosome and translation related Glyma transcript models having very low TE values (TE< = 0.1) in at least one of the three developmental stages. (XLSX)

S7 File Comparison of control RNA and ribosome profiling data alignments to all protein coding sequences of the chloroplast genome. (XLSX)

## References

[1] Kawaguchi R, Girke T, Bray EA, Bailey-Serres J. Differential mRNA translation contributes to gene regulation under non-stress and dehydration stress conditions in *Arabidopsis thaliana*. Plant J. 2004; 38: 823–839. doi: 10.1111/j.1365-313X.2004.02090.x 15144383 10.1111/j.1365-313X.2004.02090.x

[2] Juntawong P, Girke T, Bazin J, Bailey-Serres J. Translational dynamics revealed by genome-wide profiling of ribosome footprints in Arabidopsis. Proc. Natl. Acad. Sci. USA. 2014; 111: E203–E212. doi: 10.1073/pnas.1317811111 24367078 10.1073/pnas.1317811111 PMC3890782

[3] Mustroph A, Zanetti ME, Jang CJH, Holtan HE, Repetti PP, Galbraith DW, et al Profiling translatomes of discrete cell populations resolves altered cellular priorities during hypoxia in Arabidopsis. Proc. Natl. Acad. Sci. USA. 2009; 106: 18843–18848. doi: 10.1073/pnas.0906131106 19843695 10.1073/pnas.0906131106 PMC2764735

[4] Ingolia NT, Ghaemmaghami S, Newman JR, Weissman JS. Genome-wide analysis in vivo of translation with nucleotide resolution using ribosome profiling. Science. 2009; 324: 218–223. doi: 10.1126/science.1168978 19213877 10.1126/science.1168978 PMC2746483

[5] Ingolia NT, Brar GA, Rouskin S, McGeachy AM, Weissman JS. The ribosome profiling strategy for monitoring translation in vivo by deep sequencing of ribosome-protected mRNA fragments. Nat. Protoc. 2012; 7: 1534–1550. doi: 10.1038/nprot.2012.086 22836135 10.1038/nprot.2012.086 PMC3535016

[6] Gerashchenko MV, Lobano AV, Gladyshev VN. Genome-wide ribosome profiling reveals complex translational regulation in response to oxidative stress. Proc. Natl. Acad. Sci. USA. 2012; 109: 17394–17399. doi: 10.1073/pnas.1120799109 23045643 10.1073/pnas.1120799109 PMC3491468

[7] Jiao Y, Meyerowitz EM. Cell-type specific analysis of translating RNAs in developing flowers reveals new levels of control. Mol. Syst. Biol. 2010; 6: 419 doi: 10.1038/msb.2010.76 20924354 10.1038/msb.2010.76 PMC2990639

[8] Mustroph A, Bailey-Serres J. The Arabidopsis translatome cell-specific mRNA atlas: Mining suberin and cutin lipid monomer biosynthesis genes as an example for data application. Plant Signal Behav. 2010; 5: 320–324. 20220312 10.4161/psb.5.3.11187 PMC2881290

[9] Ingolia NT. Genome-wide translational profiling by ribosome footprinting. Methods Enzymol. 2010; 470: 119–142. doi: 10.1016/S0076-6879(10)70006-9 20946809 10.1016/S0076-6879(10)70006-9

[10] Ingolia NT. Ribosome profiling: new views of translation, from single codons to genome scale. Nat Rev Genet. 2014; 15: 205–213. doi: 10.1038/nrg3645 24468696 10.1038/nrg3645

[11] Jones SI, Gonzalez DO, Vodkin L. Flux of transcript patterns during soybean seed development. BMC Genomics. 2010; 11: 136 doi: 10.1186/1471-2164-11-136 20181280 10.1186/1471-2164-11-136 PMC2846912

[12] Jones SI, Vodkin L. Using RNA-Seq to profile soybean seed development from fertilization to maturity. PLoS One. 2013; 8: e59270 doi: 10.1371/journal.pone.0059270 23555009 10.1371/journal.pone.0059270 PMC3598657

[13] Meinke DW, Chen J, Beachy RN. Expression of storage-protein genes during soybean seed development. Planta. 1981; 153: 130–139. doi: 10.1007/BF00384094 24276763 10.1007/BF00384094

[14] Harada JJ, Barker SJ, Goldberg RB. Soybean b-conglycinin genes are clustered in several DNA regions and are regulated by transcriptional and posttranscriptional processes. Plant Cell. 1989; 1: 415–425. doi: 10.1105/tpc.1.4.415 2562562 10.1105/tpc.1.4.415 PMC159773

[15] Walling L, Drews GN, Goldberg RB. Transcriptional and posttranscriptional regulation of soybean seed protein mRNA levels. Proc Natl AcadSci USA. 1986; 83: 2123–2127. 10.1073/pnas.83.7.2123 PMC323243 16593677

[16] Schmidt MA, Barbazuk WB, Sandford M, May G, Song Z, Zhou W, et al Silencing of soybean seed storage proteins results in a rebalanced protein composition preserving seed protein content without major collateral changes in the metabolome and transcriptome. Plant Physiol. 2011; 156: 330–345. doi: 10.1104/pp.111.173807 21398260 10.1104/pp.111.173807 PMC3091051

[17] Md Shamimuzzaman, Vodkin L. Identification of soybean seed developmental stage-specific and tissue-specific miRNA targets by degradome sequencing. BMC Genomics. 2012; 13: 310 doi: 10.1186/1471-2164-13-310 22799740 10.1186/1471-2164-13-310 PMC3410764

[18] Schmutz J, Cannon SB, Schlueter J, Ma J, Mitros T, Nelson W, et al Genome sequence of the palaeopolyploid soybean. Nature. 2010; 463: 178–183. doi: 10.1038/nature08670 20075913 10.1038/nature08670

[19] Zanetti ME, Chang IF, Gong F, Galbraith DW, Bailey-Serres J. Immunopurification of polyribosomal complexes of Arabidopsis for global analysis of gene expression. Plant Physiol. 2005; 138: 624–635. doi: 10.1104/pp.105.059477 15955926 10.1104/pp.105.059477 PMC1150383

[20] Langmead B, Trapnell C, Pop M, Salzberg SL. Ultrafast and memory-efficient alignment of short DNA sequences to the human genome. Genome Biol. 2009; 10: R25 doi: 10.1186/gb-2009-10-3-r25 19261174 10.1186/gb-2009-10-3-r25 PMC2690996

[21] Anders S. and Huber W.. 2010 Differential expression analysis for sequence count data. Genome Biol. 11:R106 doi: 10.1186/gb-2010-11-10-r106 20979621 10.1186/gb-2010-11-10-r106 PMC3218662

[22] Mortazavi A, Williams BA, McCue K, Schaeffer L, Wold B. Mapping and quantifying mammalian transcriptomes by RNA-Seq. Nat Methods. 2008; 5: 621–628. doi: 10.1038/nmeth.1226 18516045 10.1038/nmeth.1226 PMC13303166

[23] Li H, Handsaker B, Wysoker A, Fennell T, Ruan J, Homer N, et al The sequence alignment/map format and SAMtools. 2009; Bioinformatics 25: 2078–2079. doi: 10.1093/bioinformatics/btp352 19505943 10.1093/bioinformatics/btp352 PMC2723002

[24] Robinson JT, Thorvaldsdóttir H, Winckler W, Guttman M, Lander ES, Getz G, et al Integrative genomics viewer. 2011; Nature Biotechnology 29: 24–26. doi: 10.1038/nbt.1754 21221095 10.1038/nbt.1754 PMC3346182

[25] Goodstein DM, Shu S, Howson R, Neupane R, Hayes RD, Fazo J, et al Phytozome: a comparative platform for green plant genomics. Nucleic Acids Res. 2012; 40: D1178–D1186. doi: 10.1093/nar/gkr944 22110026 10.1093/nar/gkr944 PMC3245001

[26] Saeed AI, Sharov V, White J, Li J, Liang W, Bhagabati N, et al TM4: a free, open source system for microarray data management and analysis. Biotechniques. 2003; 34: 374–378. 12613259 10.2144/03342mt01

[27] Shewry RP, Napler MA, Tatham AS. Seed storage proteins: structures and biosynthesis. Plant Cell 1995; 7:945–956. doi: 10.1105/tpc.7.7.945 7640527 10.1105/tpc.7.7.945 PMC160892

[28] Goldberg RB, Hoschek G, Vodkin LO. An insertion sequence blocks the expression of a soybean lectin gene. Cell. 1983; 33: 465–475. 6190570 10.1016/0092-8674(83)90428-2

[29] Vodkin LO, Rhodes PR, Goldberg RB 1983. A lectin gene insertion has the structural features of a transposable element. Cell 1983; 34: 1023–1031. 6313203 10.1016/0092-8674(83)90560-3

[30] Locascio A, Roig-Villanova I, Bernardi J, Varotto S. Current perspectives on the hormonal control of seed development in Arabidopsis and maize: a focus on auxin. Frontiers in Plant Sci. 2014; 5:1–22. 10.3389/fpls.2014.00412 PMC4142864 25202316

[31] Bazan J, Baerenfaller K, Gosai SJ, Gregory BD, Crespi M, Bailey-Serres J. Global anaylsis of ribosome-associated noncoding RNAs unveils new modes of translational regulation. Proc. Natl. Acad. Sci. USA. 2017; 146:e10018–e10027. 10.1073/pnas.1708433114 PMC5699049 29087317

[32] Lei L, Shi J, Chen J, Zhang M, Sun S, Xie S, et al Ribosome profiling reveals dynamic translational landscape in maize seedlings under drought stress. 2015; 84: 1206–1218. 10.1111/tpj.13073 26568274

[33] Coate JE, Bar H, and Doyle JJ. Extensive translational regulation of gene expression in an allopolyploid (*Glycine dolichocarpa*). Plant Cell. 2014; 26: 136–150. doi: 10.1105/tpc.113.119966 24488964 10.1105/tpc.113.119966 PMC3963564

[34] Bai B, Peviani A, van der Horst S, Gamm M, Snel B, Bentsink L, et al Extensive translational regulation during seed germination revealed by polysomal profiling. New Phytol. 2017; 214:233–244. doi: 10.1111/nph.14355 27935038 10.1111/nph.14355 PMC5347915

[35] Goldberg RB, Hosche G, Ditt GS, Breidenbach RW. Developmental regulation of cloned superabundant embryo mRNAs in soybean. Dev. Biol. 1981; 83: 218–231. 6113180 10.1016/0012-1606(81)90468-1

[36] Nielsen N. The structure and complexity of the 11S polypeptides in soybean. JOACS. 1985; 62:1680–1686.

[37] Siezen RJ, Leunissen JAM. Subtilases: The superfamily of subtilisin-like serine proteases. Protein Sci. 1997; 6: 501–523. doi: 10.1002/pro.5560060301 9070434 10.1002/pro.5560060301 PMC2143677

[38] Grudkowska M, Zagdanska B. Multifunctional role of plant cysteine proteinases. ActaBiochim Pol. 2004; 51: 609–624. 15448724

[39] Papastoitsis G, Wilson KA. Initiation of the Degradation of the Soybean Kunitz and Bowman-Birk Trypsin Inhibitors by a Cysteine Protease. Plant Physiol. 1991; 96: 1086–1092. 16668302 10.1104/pp.96.4.1086 PMC1080897

[40] Qi X, Wilson KA, Tan-Wilson AL. Characterization of the major protease involved in the soybean β-conglycinin storage protein mobilization. Plant Physiol. 1992; 99: 725–733. 16668946 10.1104/pp.99.2.725 PMC1080525

[41] Liu X, Zhang Z, Barnaby N, Wilson KA, Tan-Wilson A. Soybean subtilisin-like protease involved in initiating storage protein degradation. Seed Sci. Res. 2001; 11: 55–68.

[42] Beilinson V, Moskalenko OV, Livingstone DS, Reverdatto SV, Jung R, Nielsen NC. Two subtilisin-like proteases from soybean. Physiol Plant. 2002; 115: 585–597. 12121465 10.1034/j.1399-3054.2002.1150413.x

[43] Zakharov A, Carchilan M, Stepurina T, Rotari V, Wilson K, Vaintraub I. A comparative study of the role of the major proteinases of germinated common bean (Phaseolus vulgaris L.) and soybean (Glycine max (L.) Merrill) seeds in the degradation of their storage proteins. J Exp Bot. 2004; 55: 2241–2249. doi: 10.1093/jxb/erh247 15333645 10.1093/jxb/erh247

[44] Youle RJ, Huang AHC. Occurrence of low molecular weight and high cysteine containing albumin storage proteins in oilseed of diverse species. Amer J. Bot. 1981; 68:44–48.

[45] Moreno FJ, Clememte A. 2S albumin storage proteins: What makes them food allergens? Open Biochem J. 2008; 2:16–28. doi: 10.2174/1874091X00802010016 18949071 10.2174/1874091X00802010016 PMC2570561

[46] Otegui MS, Herder R, Schulze J, Jung R, Staehelin LA. The proteolytic processing of seed sstorage proteins in Arabidopsis embryo cells starts in the multivesicular bodies. Plant Cell 2006; 18:2567–2581. doi: 10.1105/tpc.106.040931 17012602 10.1105/tpc.106.040931 PMC1626608

[47] Mylne JS, Hara-Nishimura K, Rosengren KJ. Seed storage albumins: biosynthesis, trafficking and structures. Funct Plant Biol. 2014; 41:671–677. 10.1071/FP14035 32481022

[48] Zabala G, Vodkin LO. 2005 The *wp* mutation of *Glycine max* carries a gene-fragment-rich transposon of the CACTA superfamily. Plant Cell 17: 2619–2632. doi: 10.1105/tpc.105.033506 16141454 10.1105/tpc.105.033506 PMC1242261

[49] Asano K. Why is start codon selection so precise in eukaryotes? Translation 2014; 2: e28387 doi: 10.4161/trla.28387 26779403 10.4161/trla.28387 PMC4705826

[50] Hinnebusch AG, Ivanov IP, Sonenberg N. Translational control by 5'-untranslated regions of eukaryotic mRNAs. Science 2016; 352: 1413–1416. doi: 10.1126/science.aad9868 27313038 10.1126/science.aad9868 PMC7422601

[51] Hinnebusch AG, Dever TE, Asano K. Mechanism of translation initiation in the yeast Saccharomyces cerevisiae In Mathews MB, Sonenberg N, Hershey JWB, editors. Translational Control in Biology and Medicine, Cold Spring Harbor, NY: Cold Spring Harbor Lab Press; 2007 pp. 225–268.

[52] Sonenberg N, Hinnebusch AG (2009). Regulation of translation initiation in eukaryotes: mechanisms and biological targets. Cell 2009; 136:731–745. doi: 10.1016/j.cell.2009.01.042 19239892 10.1016/j.cell.2009.01.042 PMC3610329

[53] Smaczniak C, Immink RGH, Angenent GC, Kaufmann K. Developmental and evolutionary diversity of plant MADS domainfactors: insights from recent studies. Development. 2012; 139: 3081–3098. doi: 10.1242/dev.074674 22872082 10.1242/dev.074674

[54] Huang F, Xu GL, Chi YJ, Liu HC, Xue Q, Zhao TJ, et al A soybean MADS-box protein modulates floral organ numbers, petal identity and sterility. BMC Plant Biol. 2014; 14: 89 doi: 10.1186/1471-2229-14-89 24693922 10.1186/1471-2229-14-89 PMC4021551

[55] Wei B, Zhang RZ, Guo JJ, Liu DM, Li AL, Fan RC. Genome-wide analysis of the MADS-box gene family in *Brachypodiumdistachyon*. PLoS One. 2014; 9: e84781 doi: 10.1371/journal.pone.0084781 24454749 10.1371/journal.pone.0084781 PMC3890268

[56] Liu W, Han X, Zhan G, Zhao Z, Feng Y, Wu C. A novel sucrose-regulatory MADS-Box transcription factor GmNMHC5 promotes root development and nodulation in soybean (*Glycine max* [L.] Merr.). Int. J. Mol. Sci. 2015; 16:20657–20673. doi: 10.3390/ijms160920657 26404246 10.3390/ijms160920657 PMC4613224

[57] Kumar G, Arya P, Gupta K, Randhawa V, Acharya V, Singh AK. Comparative phylogenetic analysis and transcriptional profiling of MADS-box gene family identified *DAM* and *FLC*-like genes in apple (*Malus* x *domestica*). Scientific Reports. 2016; 6: 20695 doi: 10.1038/srep20695 26856238 10.1038/srep20695 PMC4746589

[58] Fan CM, Wang X, Wang YW, Hu RB, Zhang XM. Genome-Wide Expression Analysis of Soybean MADS Genes Showing Potential Function in the Seed Development. PloS one. 2013; 8: e62288 doi: 10.1371/journal.pone.0062288 23638026 10.1371/journal.pone.0062288 PMC3640087

[59] Andrianopoulos A, Kourambas S, Sharp JA, Davis MA, Hynes MJ. Characterization of the *Aspergillus nidulans* nmrA gene involvd in nitrogen metabolite repression. 1998. J Bacteriol. 1998;180:1973–1977. 9537404 10.1128/jb.180.7.1973-1977.1998 PMC107119

[60] Rainbird RN, Thorne JH, Hardy WF. Role of amides, amino acids, and ureides in the nutrition of developing soybean seeds. Plant Physiol. 1984; 74:329–334. 16663418 10.1104/pp.74.2.329 PMC1066678

[61] Md Shamimuzzaman, Vodkin L. Transcription factors and glyoxylate cycle genes prominent in the transition of soybean cotyledons to the first functional leaves of the seedling. Funct Integr Genomics. 2014; 14: 683–696. doi: 10.1007/s10142-014-0388-x 25070765 10.1007/s10142-014-0388-x

[62] Shi Z, Fujii K, Kovary KM, Genuth NR, Rost HL, Teurel MN, et al Heterogeneous ribosomes preferentially translate distinct subpools of mRNAs genome-wide. Molecular Cell 2017; 67:1–13. 28625553 10.1016/j.molcel.2017.05.021 PMC5548184

[63] Chang S, Wang Y, Lu J, Gai J, Li J, Chu P, et al The mitochondrial genome of soybean reveals complex genome structures and gene evolution at intercellular and phylogenetic levels. PLoS One. 2013; 8: e56502 doi: 10.1371/journal.pone.0056502 23431381 10.1371/journal.pone.0056502 PMC3576410

[64] Saski C, Lee S-B, Daniell H, Wood TC, Tomkins J, Kim H-G, et al Complete chloroplast genome sequence of *Glycine max* and comparative analyses with other legume genomes. Plant Mol. Biol. 2005; 59: 309–322. doi: 10.1007/s11103-005-8882-0 16247559 10.1007/s11103-005-8882-0

[65] Saito GY, Chang YC, Walling LL, Thomson WW. A correlation in plastid development and cytoplasmic ultrastructure with nuclear gene expression during seed ripening in soybean. New Phytol. 1989; 113:459–469.

[66] Chotewutmontri P, Barkan A. Dynamics of chloroplast translation during chloroplast differentiation in maize. PLOS Genetics 2017; 12: e1006106. 10.1371/journal.pgen.1006106 PMC4945096 27414025

[67] Goldberg RB, Barker SJ, Perez-Grau L. Regulation of gene expression during plant embryogenesis. Cell. 1989; 56: 149–160. 2643470 10.1016/0092-8674(89)90888-x

[68] Golombek S, Rolletsch H, Wobus U, Weber H. Control of storage protein accumulation during legume seed development. Plant Physiol. 2001; 158: 457–464.

[69] Le BH, Wagmaister JA, Kawashima T, Bui AQ, Harada JJ, Goldberg RB. Using genomics to study legume seed development. Plant Physiol. 2007; 144:562–74. doi: 10.1104/pp.107.100362 17556519 10.1104/pp.107.100362 PMC1914191

[70] Gutierrez L, Van Wuytswinkel O, Castelain M, Bellini C. Combined networks regulating seed maturation. Trends Plant Sci. 2007; 12: 294–300. doi: 10.1016/j.tplants.2007.06.003 17588801 10.1016/j.tplants.2007.06.003

[71] Vodkin LO. Isolation and characterization of messenger RNAs for seed lectin and Kunitz trypsin inhibitor in soybean. Plant Physiology. 1981; 68: 766–771. 16661996 10.1104/pp.68.3.766 PMC425978

[72] Philip R., Darnowski D.W., Sundararaman V., Cho M-J., Vodkin L.O. 1998 Localization of β-glucuronidase in protein bodies of transgenic tobacco seed by fusion to an amino terminal sequence of the soybean lectin gene. Plant Science 137: 191–204.

