# Supplementary figures and images for: Ribosome profiling reveals changes in translational status of soybean transcripts during immature cotyledon development
Source: PLoS One. 2018 Mar 23;13(3):e0194596. doi: 10.1371/journal.pone.0194596 (PMC5865733; doi:10.1371/journal.pone.0194596)

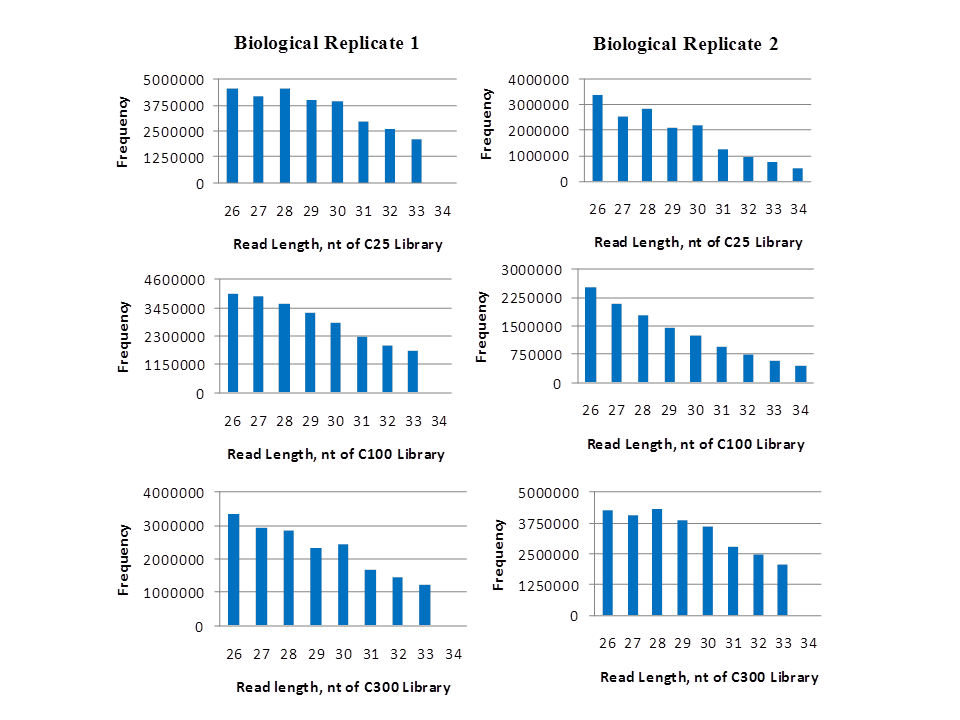

Supplement: S1 Fig — (TIF) [file pone.0194596.s001.tif]

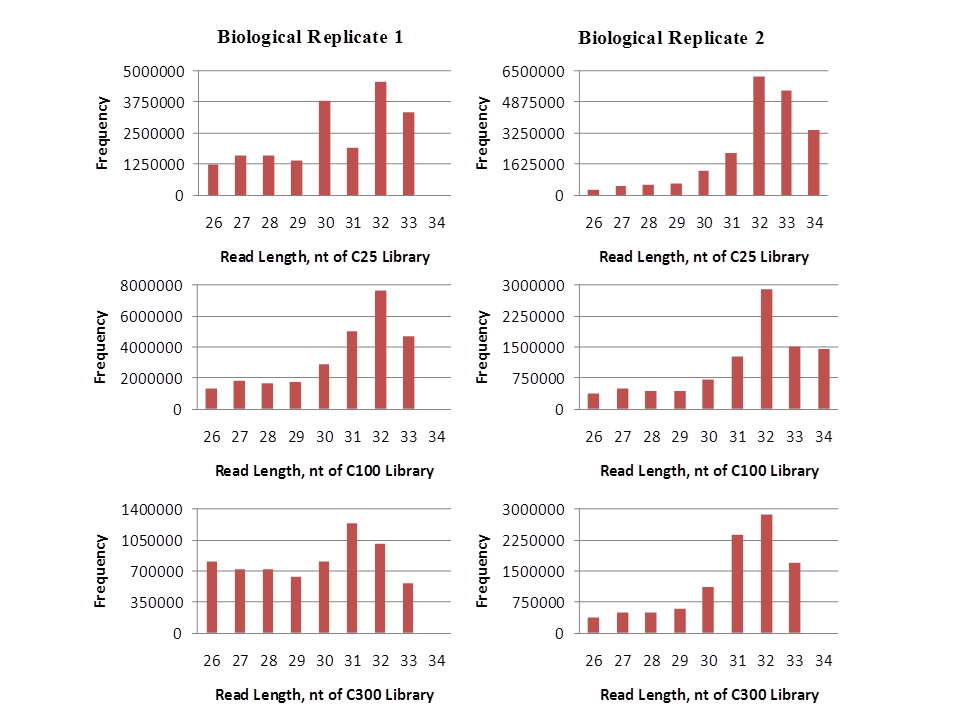

Supplement: S2 Fig — (TIF) [file pone.0194596.s002.tif]
