# Supplementary material for: Ribosome profiling reveals changes in translational status of soybean transcripts during immature cotyledon development
Source: PLoS One. 2018 Mar 23;13(3):e0194596. doi: 10.1371/journal.pone.0194596 (PMC5865733; doi:10.1371/journal.pone.0194596)
Supplement: S3 Fig — (DOCX) [file pone.0194596.s003.docx]

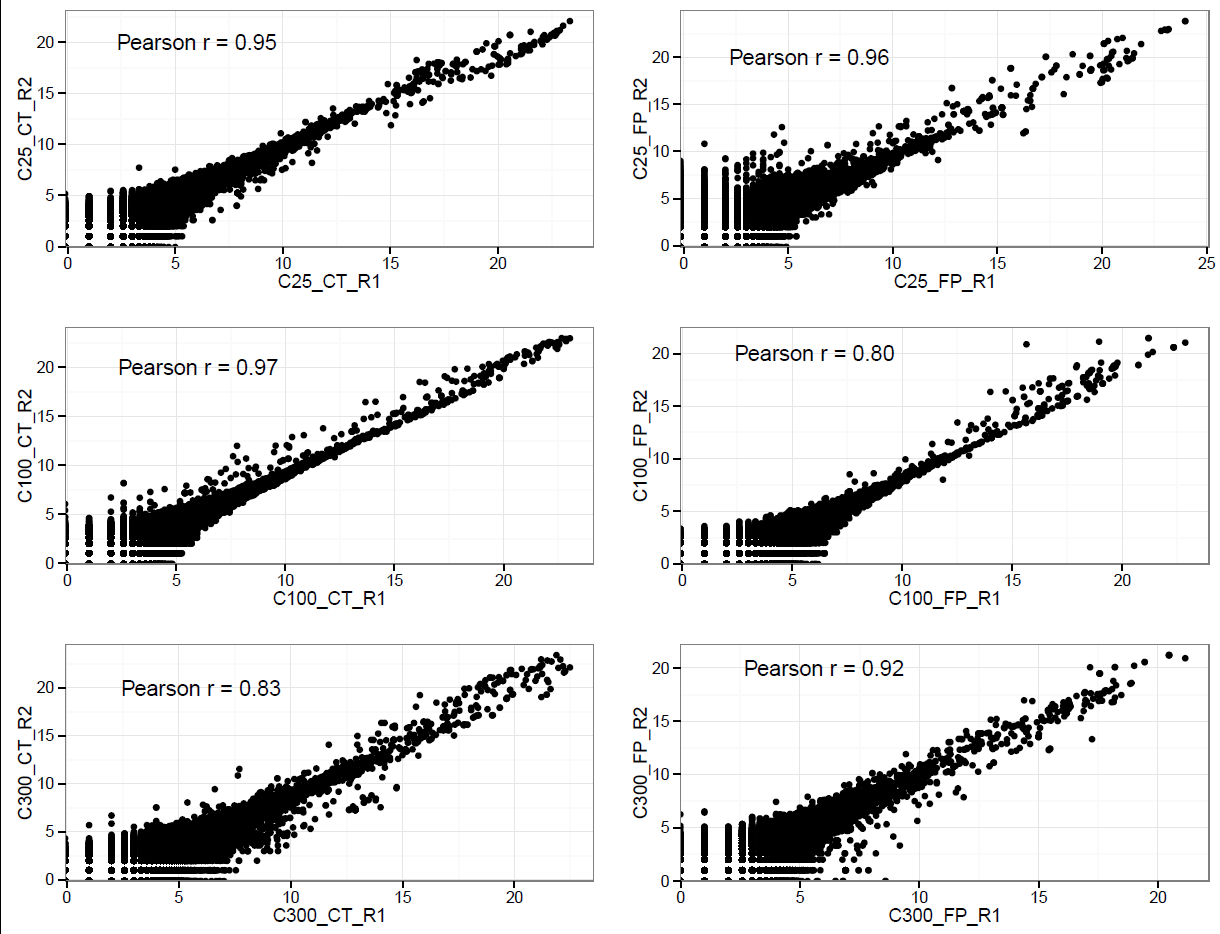


**S3 Fig. Biological replicates of cotyledon samples show reproducibility within RNA sequencing as well as ribosome profiling libraries.** The calculated Pearson correlation coefficient(r) is shown for both RNA sequencing (CT) and ribosome footprint (FP) samples. C25 denotes early stage cotyledons of 25-50 mg, C100 denotes mid stage cotyledons of 100-200 mg, and C300 denotes late stage cotyledons of 300-400 mg. R1 and R2 denote biological replicates shown on the left and right panels, respectively.
