# Supplementary material for: Ribosome profiling reveals changes in translational status of soybean transcripts during immature cotyledon development
Source: PLoS One. 2018 Mar 23;13(3):e0194596. doi: 10.1371/journal.pone.0194596 (PMC5865733; doi:10.1371/journal.pone.0194596)
Supplement: S1 Table — (DOCX) [file pone.0194596.s004.docx]

**S1 Table. Oligonucleotides used in RNA sequencing and ribosome footprint library preparation.**

| **Oligonucleotide** | **Sequence (5'-3')** |
| --- | --- |
| Upper Marker | AUGUACACGGAGUCGAGCUCAACCCGCAACGCGA-(Phos) |
| Lower Marker | AUGUACACGGAGUCGACCCAACGCGA-(Phos) |
| Linker | 1/5rApp/CTGTAGGCACCATCAAT/3ddC/ |
| RT Primer | (Phos)-AGATCGGAAGAGCGTCGTGTAGGGAAAGAGTGTAGATCTCGGTGGTCGC- |
|  | (SpC18)-CAC TCA-(SpC18)-TTCAGACGTGTGCTCTTCCGATCTATTGATGGTGCCTACAG |
| Forward Primer | AATGATACGGCGACCACCGAGATCTACAC |
| Reverse Primer 1 | CAAGCAGAAGACGGCATACGAGATAGTCGTGTGACTGGAGTTCAGACGTGTGCTCTTCCG |
| Reverse Primer 2 | CAAGCAGAAGACGGCATACGAGATACTGATGTGACTGGAGTTCAGACGTGTGCTCTTCCG |
| Reverse Primer 3 | CAAGCAGAAGACGGCATACGAGATATGCTGGTGACTGGAGTTCAGACGTGTGCTCTTCCG |
| Reverse Primer 4 | CAAGCAGAAGACGGCATACGAGATACGTCGGTGACTGGAGTTCAGACGTGTGCTCTTCCG |
| Reverse Primer 5 | CAAGCAGAAGACGGCATACGAGATAGCTGCGTGACTGGAGTTCAGACGTGTGCTCTTCCG |
| Reverse Primer 6 | CAAGCAGAAGACGGCATACGAGATATCGTAGTGACTGGAGTTCAGACGTGTGCTCTTCCG |
| Reverse Primer 7 | CAAGCAGAAGACGGCATACGAGATCGTCAGGTGACTGGAGTTCAGACGTGTGCTCTTCCG |
