# Supplementary material for: Ribosome profiling reveals changes in translational status of soybean transcripts during immature cotyledon development
Source: PLoS One. 2018 Mar 23;13(3):e0194596. doi: 10.1371/journal.pone.0194596 (PMC5865733; doi:10.1371/journal.pone.0194596)
Supplement: S2 Table — (DOCX) [file pone.0194596.s005.docx]

**S2 Table. Summary of RNA sequencing and ribosome profiling reads.**

| **Library**  **Full Name** | **Raw Reads** | **Reads Processed** | **Total Mapped Reads** | **rDNA Reads** | **Gene Mapped Reads** | **Developmental**  **Stage** | **Library**  **Type** |
| --- | --- | --- | --- | --- | --- | --- | --- |
| RP2502C_Rep1 | 89,827,739 | 54,279,162 | 126,913,215 | 107,818,311 | 19,094,904 | 25-50 mg, C25 | control RNA |
| RP2505C_Rep2 | 113,415,356 | 34,066,588 | 49,856,818 | 35,804,456 | 14,052,362 | 25-50 mg, C25 | control RNA |
| RP2502T_Rep1 | 51,640,489 | 25,036,524 | 73,403,289 | 57,158,834 | 16,244,455 | 25-50 mg, C25 | footprint RNA |
| RP2505T_Rep2 | 77,525,914 | 35,911,773 | 64,369,671 | 45,112,958 | 19,256,713 | 25-50 mg, C25 | footprint RNA |
| RP1003C_Rep1 | 49,802,836 | 38,904,291 | 104,093,065 | 82,448,249 | 21,644,816 | 100-200 mg, C100 | control RNA |
| RP1005C_Rep2 | 81,333,445 | 39,639,476 | 113,151,450 | 97,849,811 | 15,301,639 | 100-200 mg, C100 | control RNA |
| RP1003T_Rep1 | 86,277,212 | 42,644,648 | 37,878,455 | 13,125,550 | 24,752,905 | 100-200 mg, C100 | footprint RNA |
| RP1005T_Rep2 | 100,907,777 | 24,435,470 | 19,322,367 | 11,014,481 | 8,307,886 | 100-200 mg, C100 | footprint RNA |
| RP3001C_Rep1 | 49,802,836 | 44,320,765 | 72,267,735 | 52,143,859 | 20,123,876 | 300-400 mg, C300 | control RNA |
| RP3003C_Rep2 | 66,928,832 | 43,272,494 | 100,476,912 | 88,515,940 | 11,960,972 | 300-400 mg, C300 | control RNA |
| RP3001T_Rep1 | 86,277,212 | 13,734,711 | 12,283,241 | 4,184,949 | 8,098,292 | 300-400 mg, C300 | footprint RNA |
| RP3003T_Rep2 | 66,026,031 | 15,038,556 | 17,198,999 | 4,950,878 | 12,248,121 | 300-400 mg, C300 | footprint RNA |

Library name starting with RP25 refers to early stage C25 (25-50 mg seed weight range)

Library name starting with RP100 refers to mid stage C100 (100-200 mg seed weight range)

Library name starting with RP300 refers to late stage C300 (300-400 mg seed weight range)

Library name ending with C denotes total RNA sequencing libraries

Library name ending with T denotes ribosome footprint libraries

Rep, Replicate 1 or 2
