# Supplementary material for: Ribosome profiling reveals changes in translational status of soybean transcripts during immature cotyledon development
Source: PLoS One. 2018 Mar 23;13(3):e0194596. doi: 10.1371/journal.pone.0194596 (PMC5865733; doi:10.1371/journal.pone.0194596)

Summary for C25

Filtering Criteria for High TE value:
(Pval<0.05; TE>1 and FP_RPKM>=1)
Total Number of Genes: 179
Cluster1 : 23 genes
Cluster 2: 10 genes
Cluster 3:   4 genes
Cluster 4:142 genes

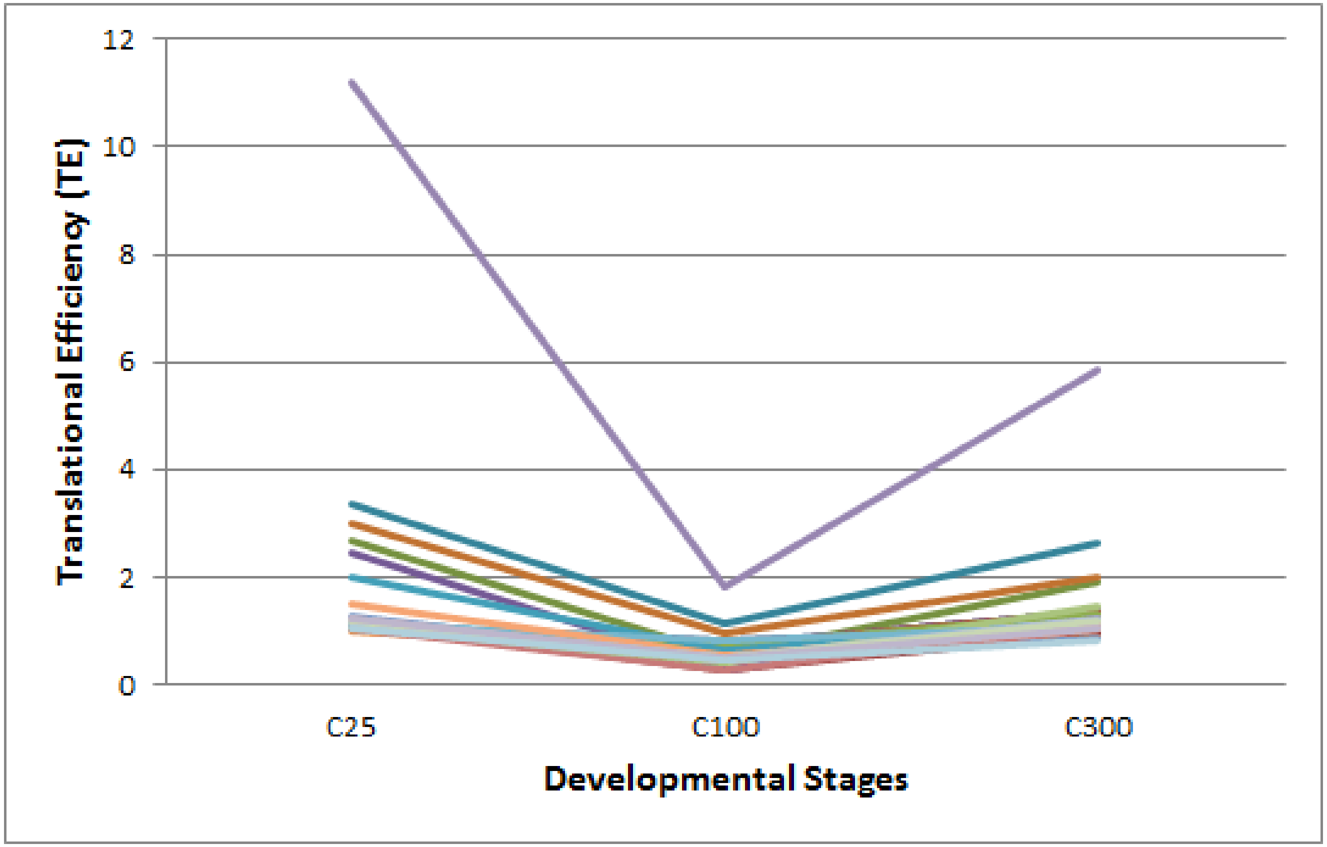

Cluster 1: 23 Genes

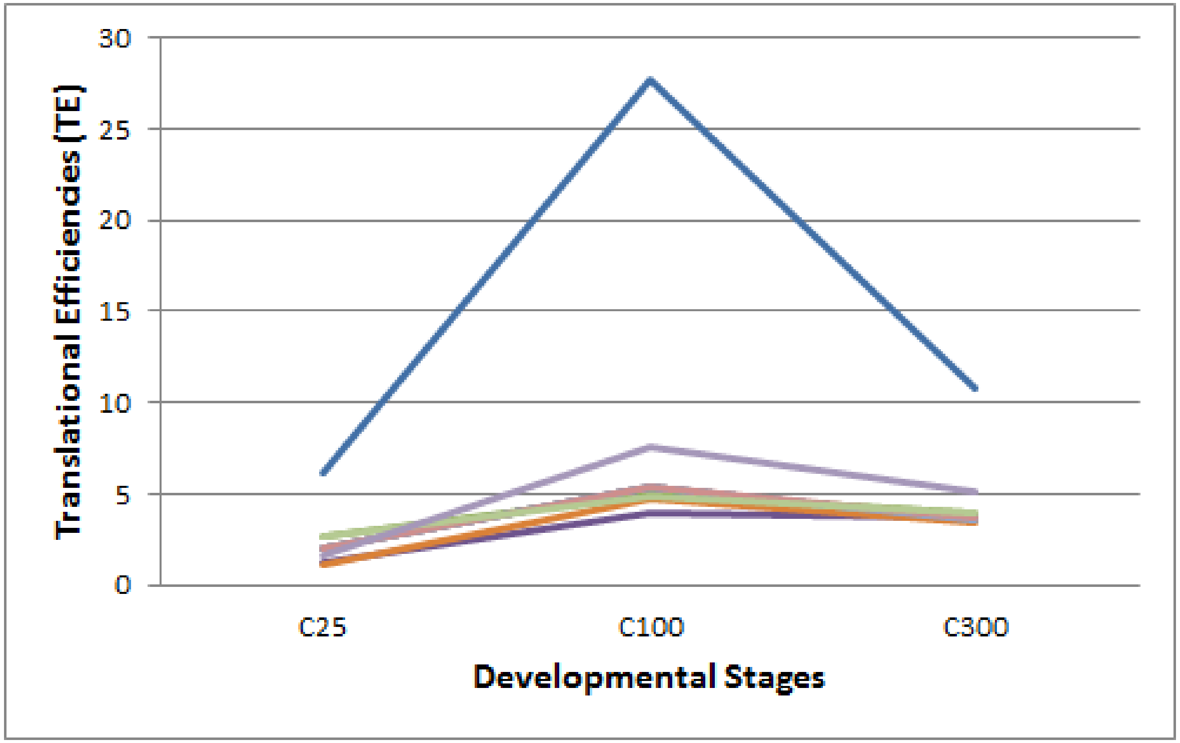

Cluster2: 10 genes

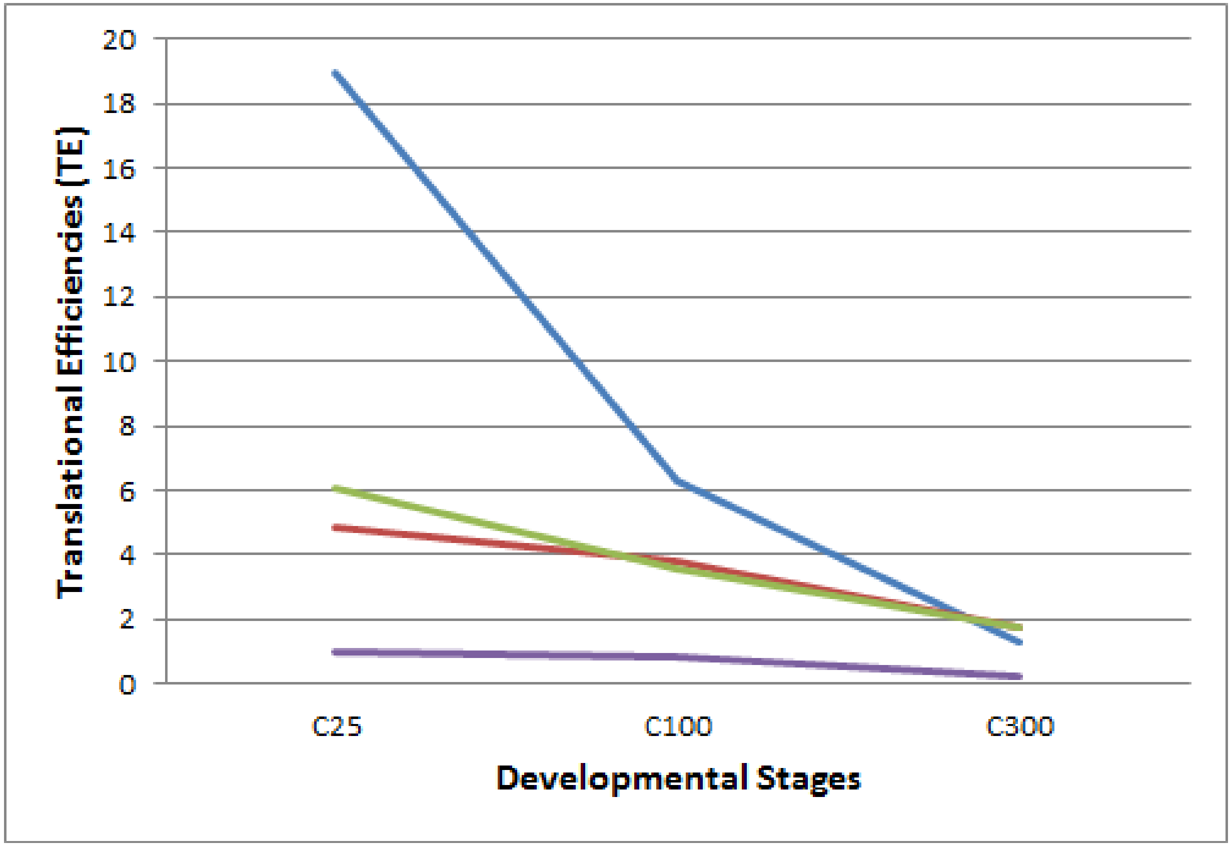

Cluster 3: 4 Genes

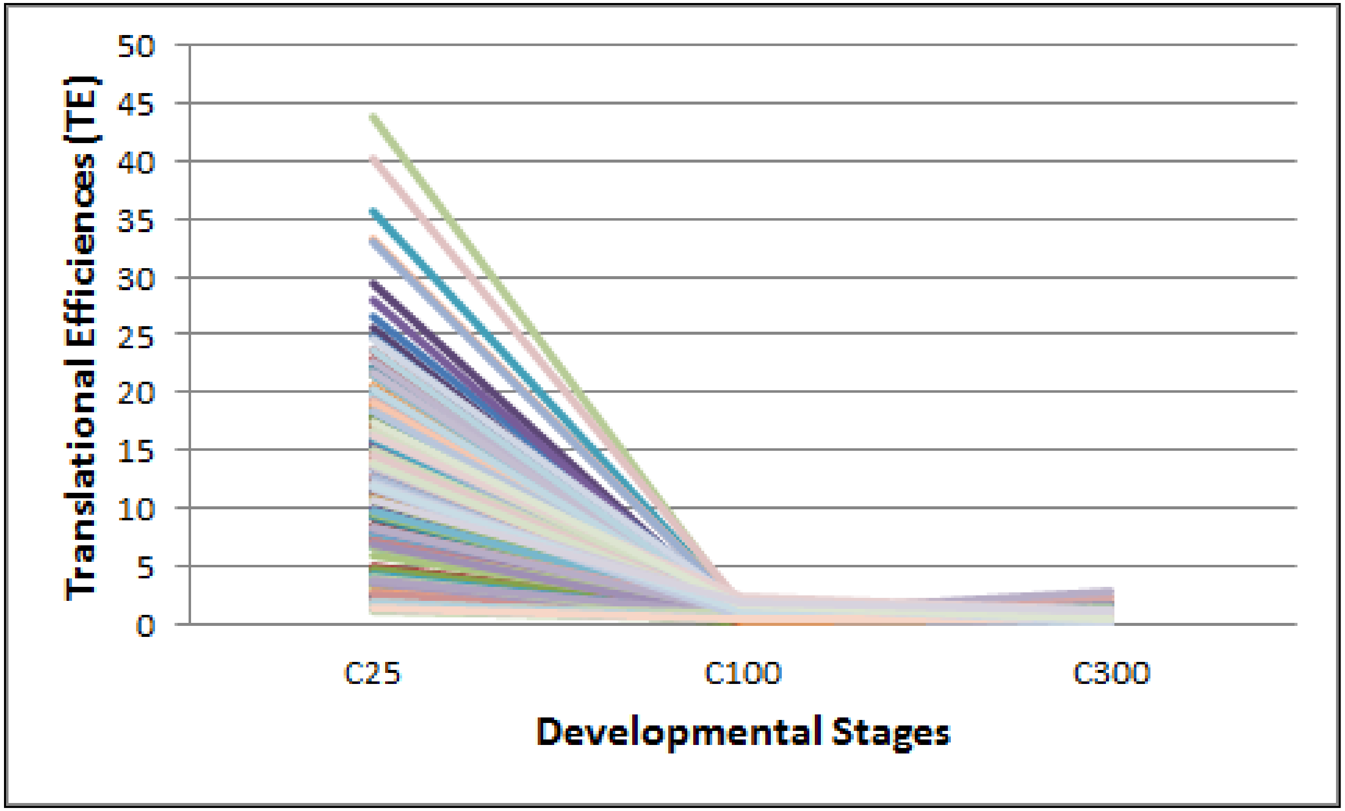

Cluster 4: 142 genes

Summary for C100

Filtering Criteria for High TE: (Pval<0.05; TE>1 and FP_RPKM>=1)

Total Number of Genes: 90

Cluster1 : 32 genes

Cluster 2: 25 genes

Cluster 3:   3 genes

Cluster 4: 30 genes

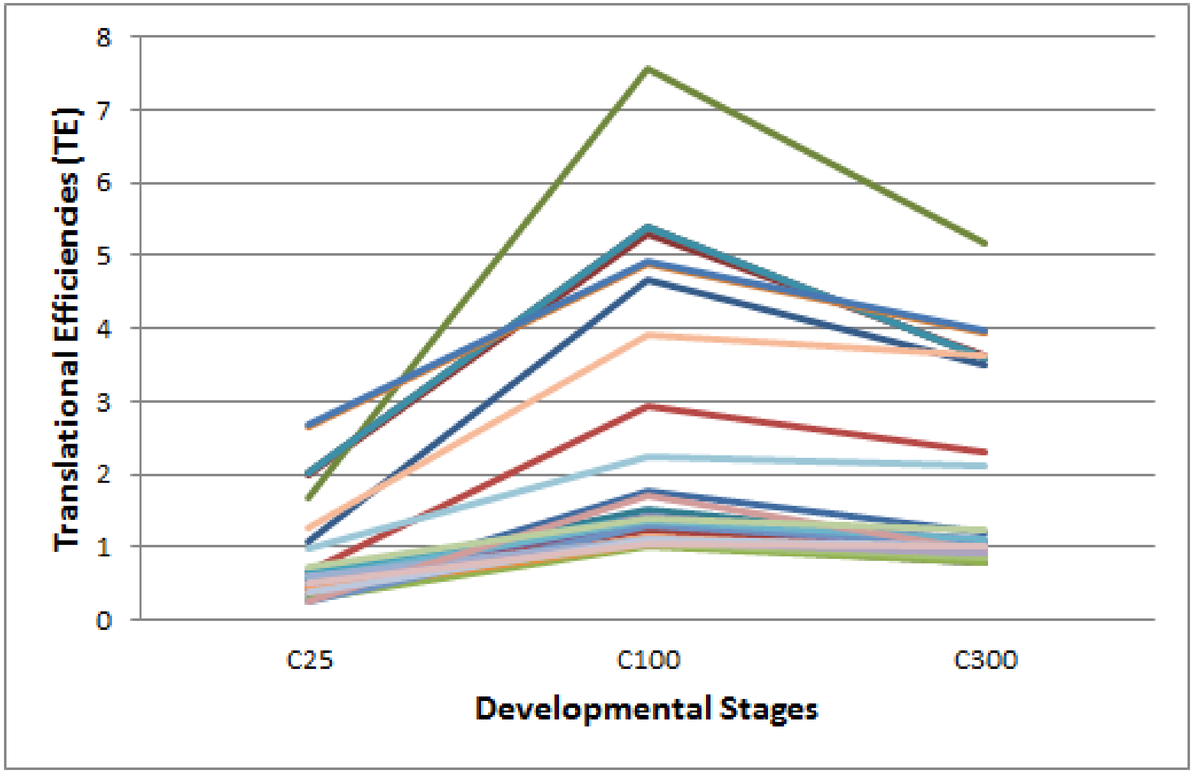

Cluster 1: 32 genes

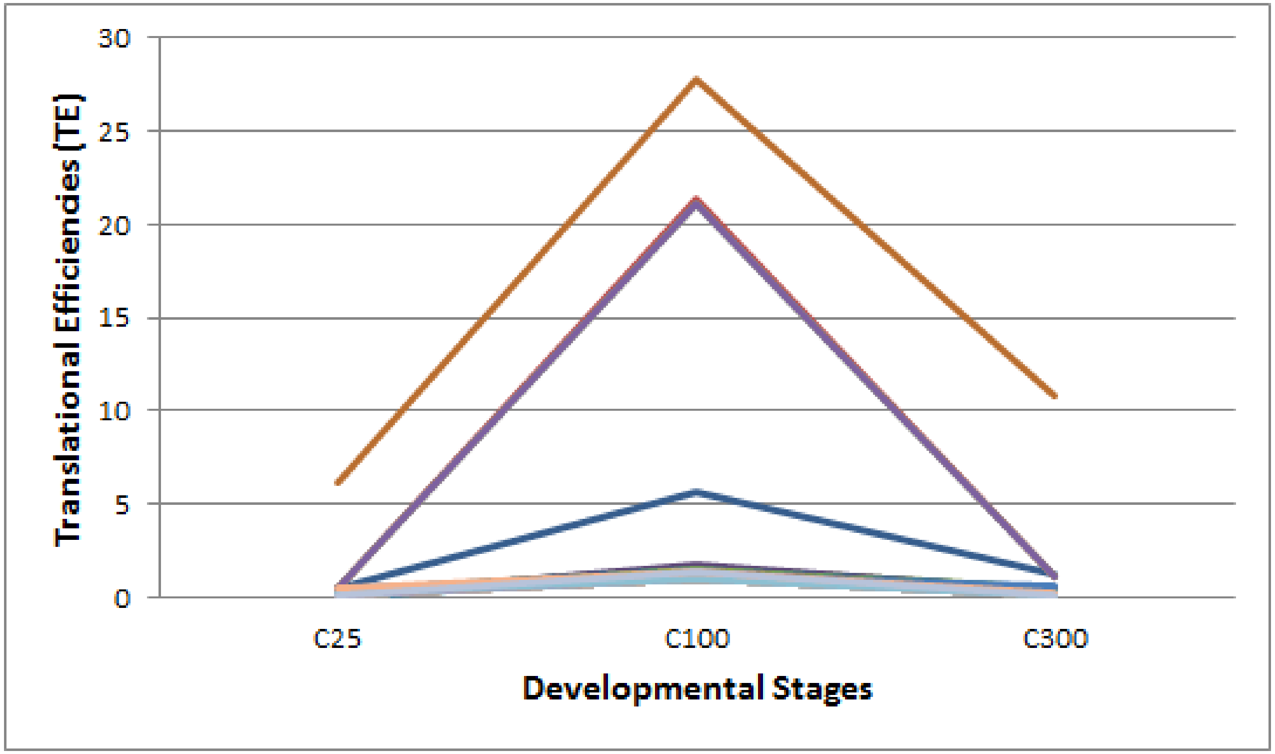

Cluster 2: 25 genes

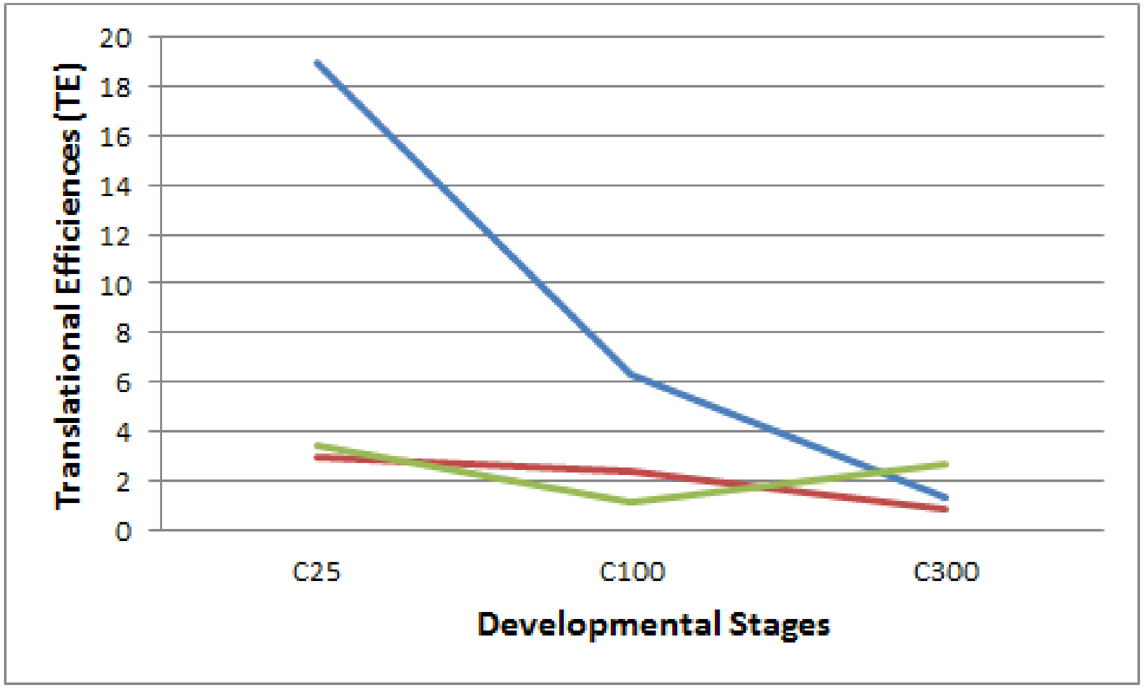

Cluster 3: 3 genes

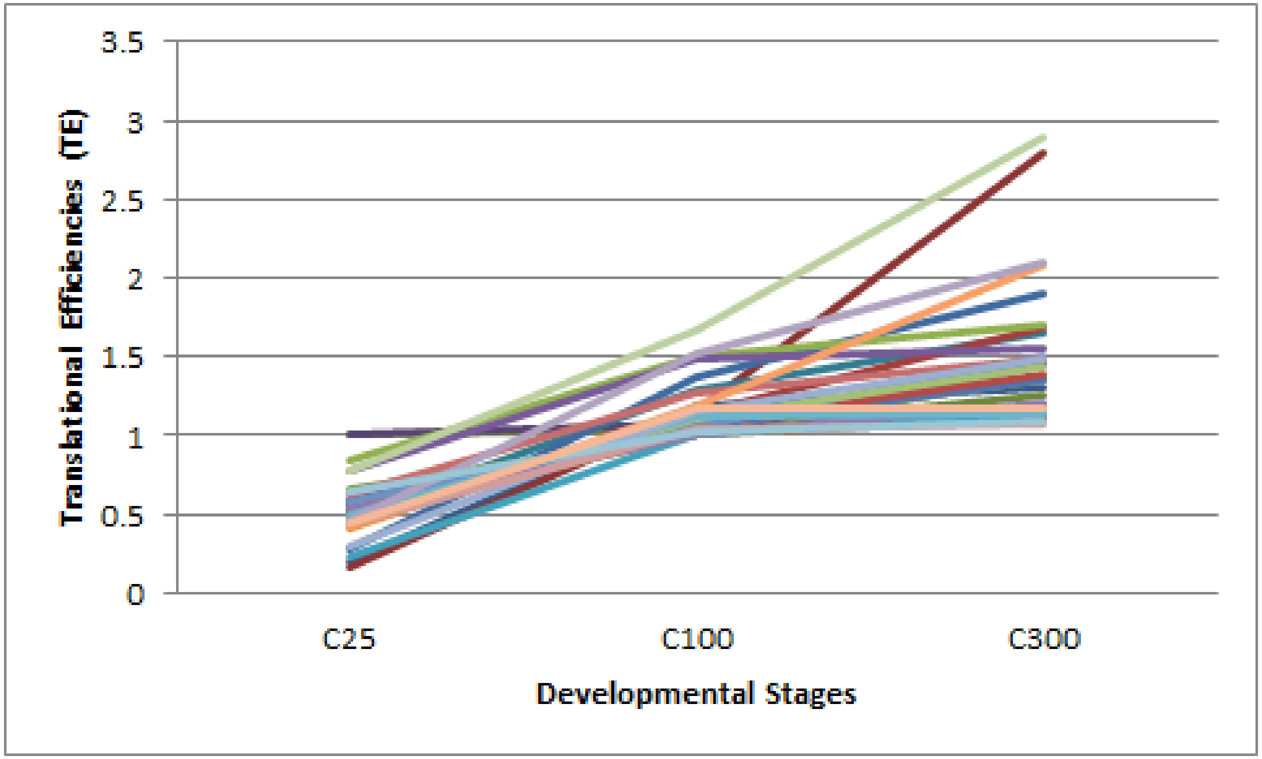

Cluster 4: 30 genes

Summary for C300

Filtering Criteria for High TE: (Pval<0.05; TE>1 and FP_RPKM>=1)

Total Number of Genes: 53

Cluster1 : 14 genes

Cluster 2:   2 genes

Cluster 3:   37 genes

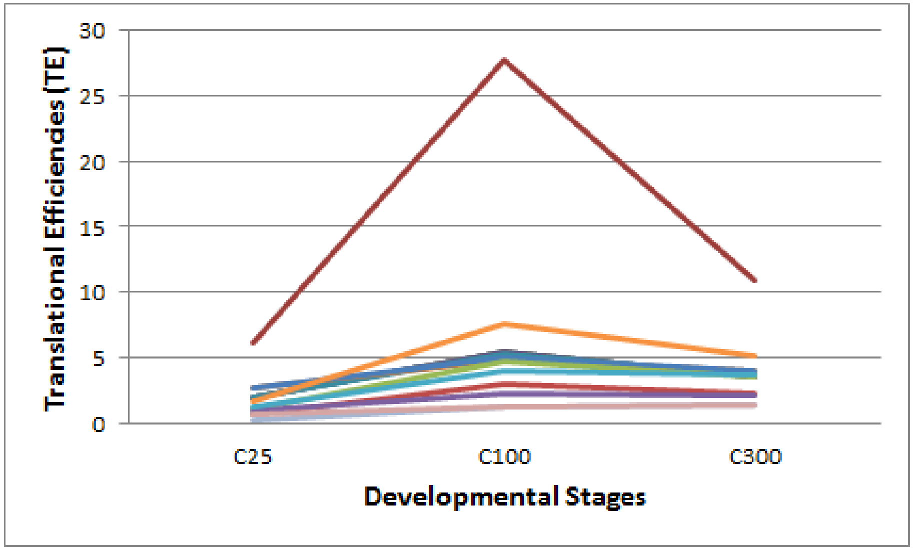
30
25
20
15
10
5
0
Translational Efficiencies (TE)
C25
C100
C300
Developmental Stages

Cluster 1: 14

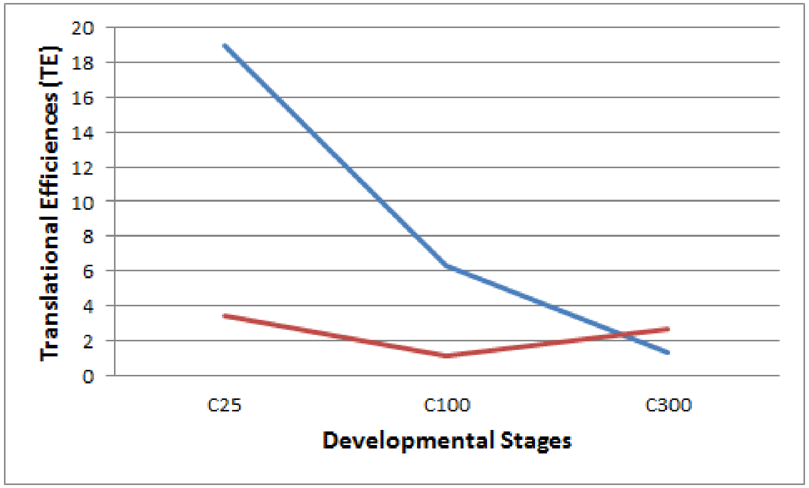

Cluster 2: 2 genes

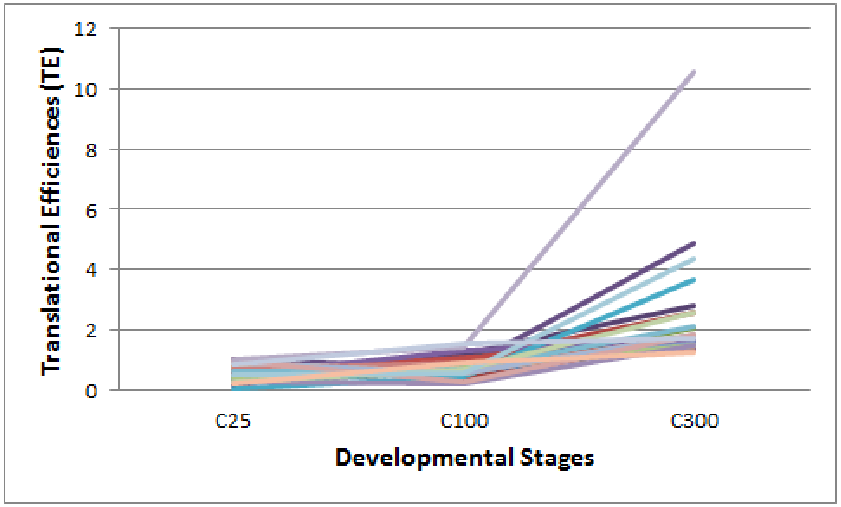

Cluster 3: 37 genes

C25_Cluster 4: 142 genes

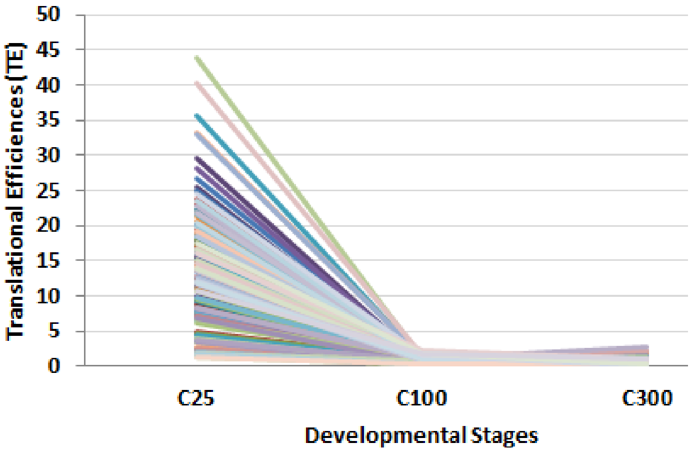

C100_Cluster 1: 32 genes

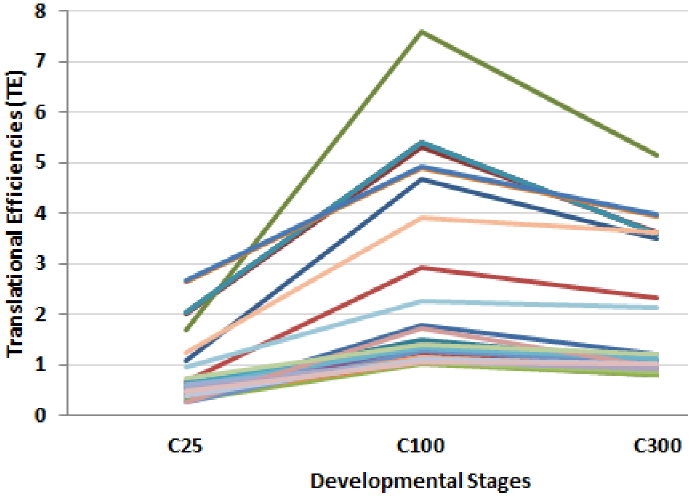

C300_Cluster 3: 37 genes

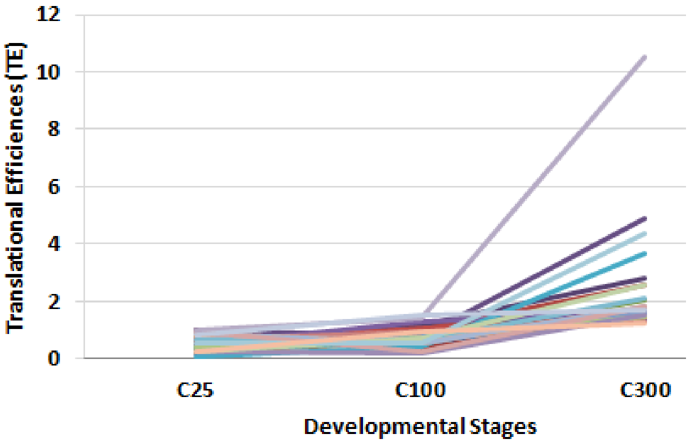

Supplement: S3 File — (PDF) [file pone.0194596.s008.pdf]
