# Supplementary material for: Ribosome profiling reveals changes in translational status of soybean transcripts during immature cotyledon development
Source: PLoS One. 2018 Mar 23;13(3):e0194596. doi: 10.1371/journal.pone.0194596 (PMC5865733; doi:10.1371/journal.pone.0194596)

Summary for C25

Filtering Criteria for Very Low TE values:

(Pval<0.05; TE<=0.1 and CT_RPKM>=10)

Total Number of Genes: 302

Cluster1 : 100 genes

Cluster 2: 88 genes

Cluster 3: 114 genes

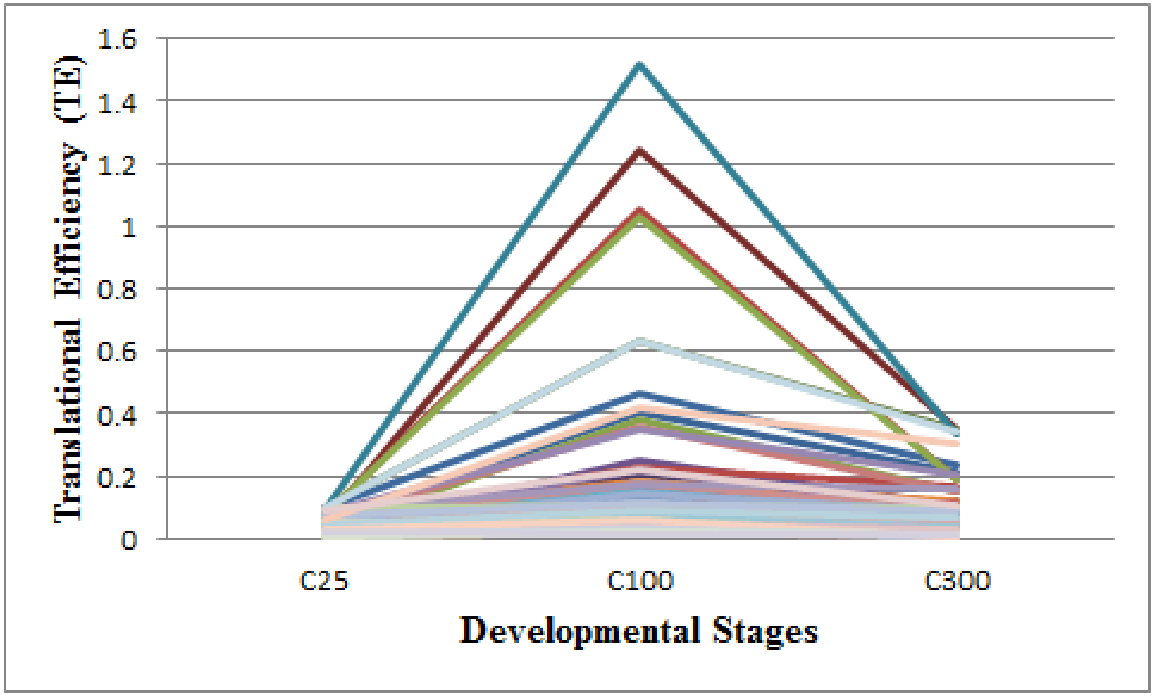

Cluster1 : 100 genes

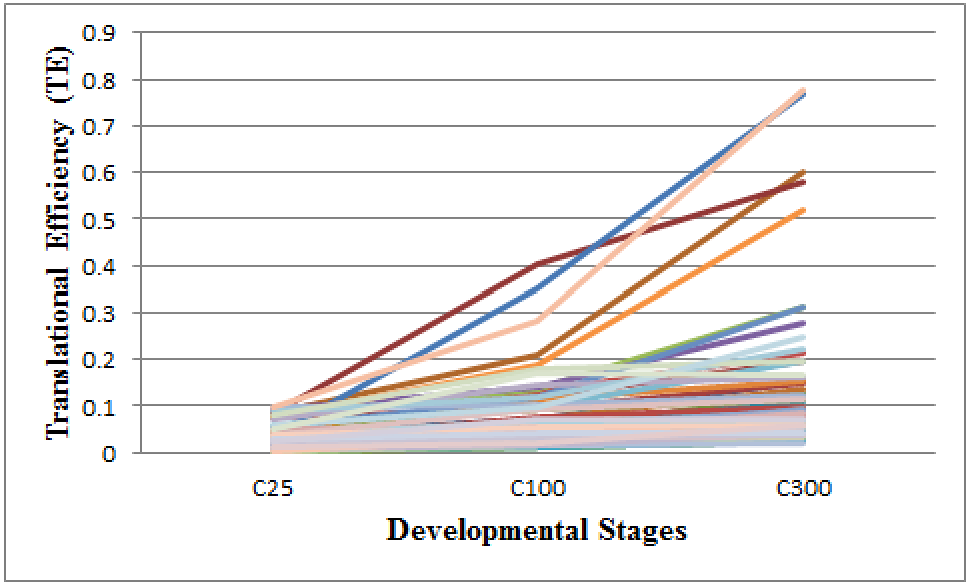

Cluster 2: 88 genes

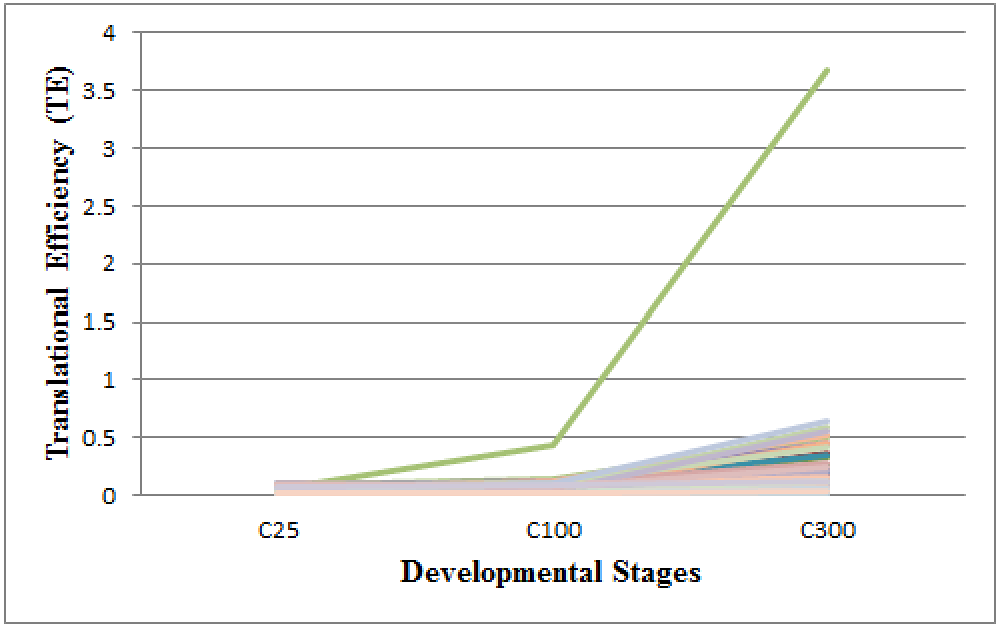

Cluster 3: 114 genes

Summary for C100

Filtering Criteria for Very Low TE Values:

(Pval<0.05; TE<=0.1 and CT_RPKM>=10)

Total Number of Genes: 218

Cluster1 : 66 genes

Cluster 2: 42 genes

Cluster 3: 110 genes

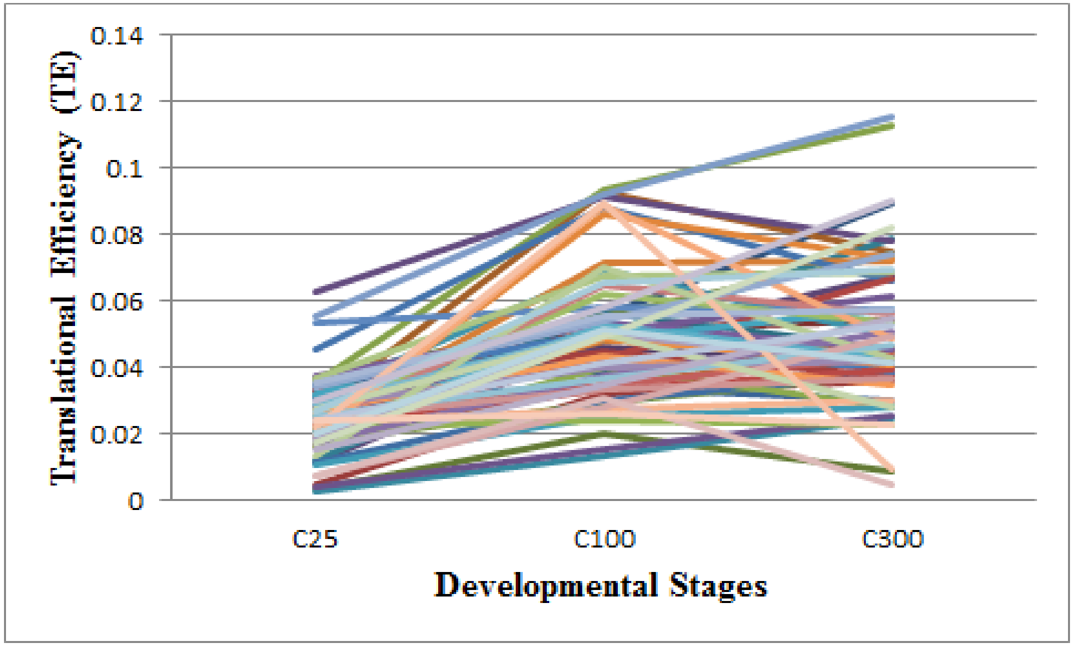
0.14
0.12
0.1
0.08
0.06
0.04
0.02
0
Translational Efficiency (TE)
C25
C100
C300
Developmental Stages

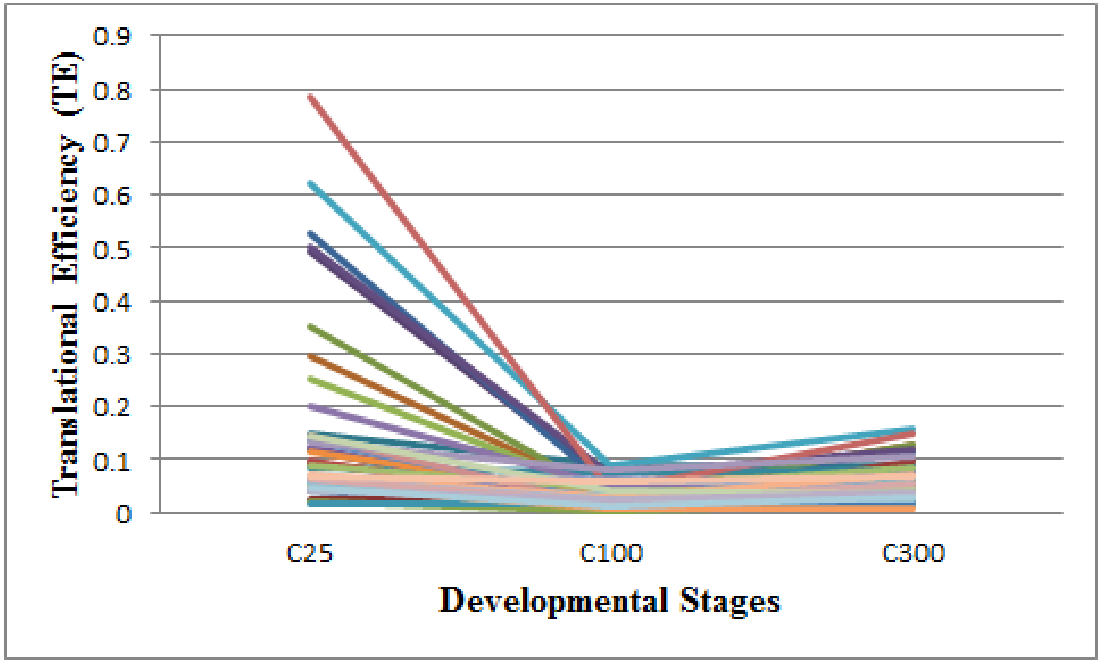

0.9
0.8
0.7
0.6
0.5
0.4
0.3
0.2
0.1
0
Translational Efficiency (TE)
C25
C100
C300
Developmental Stages

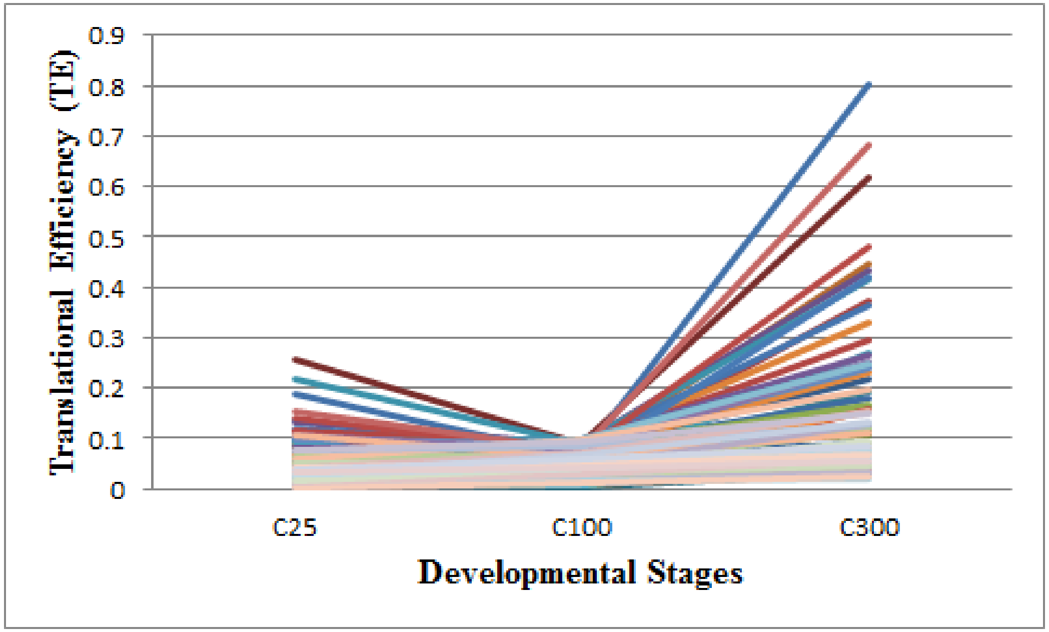

Translational Efficiency (TE)
0.9
0.8
0.7
0.6
0.5
0.4
0.3
0.2
0.1
0
C25
C100
C300
Developmental Stages

Summary for C300

Filtering Criteria for Very Low TE Values:

(Pval<0.05; TE<=0.1 and CT_RPKM>=10)

Total Number of Genes: 244

Cluster1 : 76 genes

Cluster 2: 47 genes

Cluster 3: 121 genes

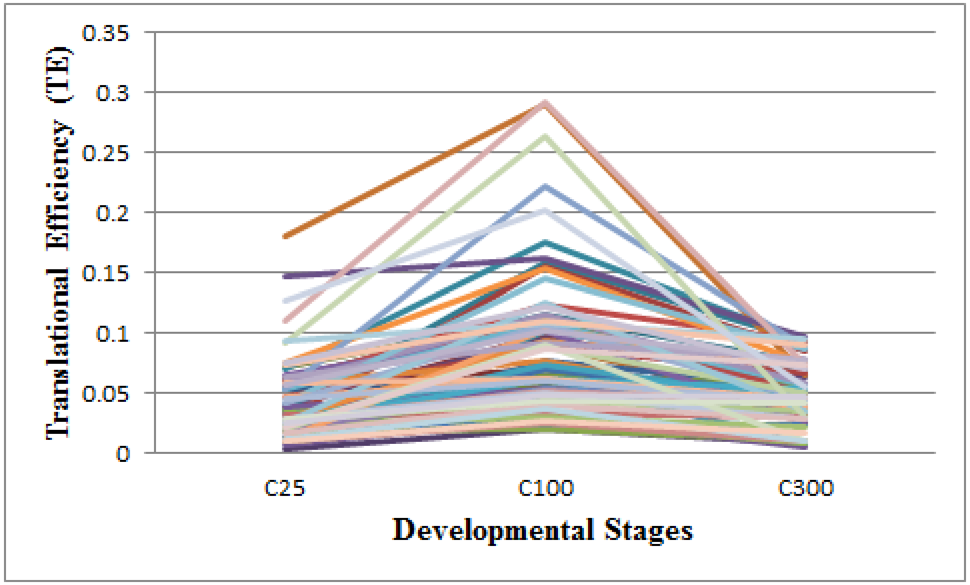

Cluster1 : 76 genes

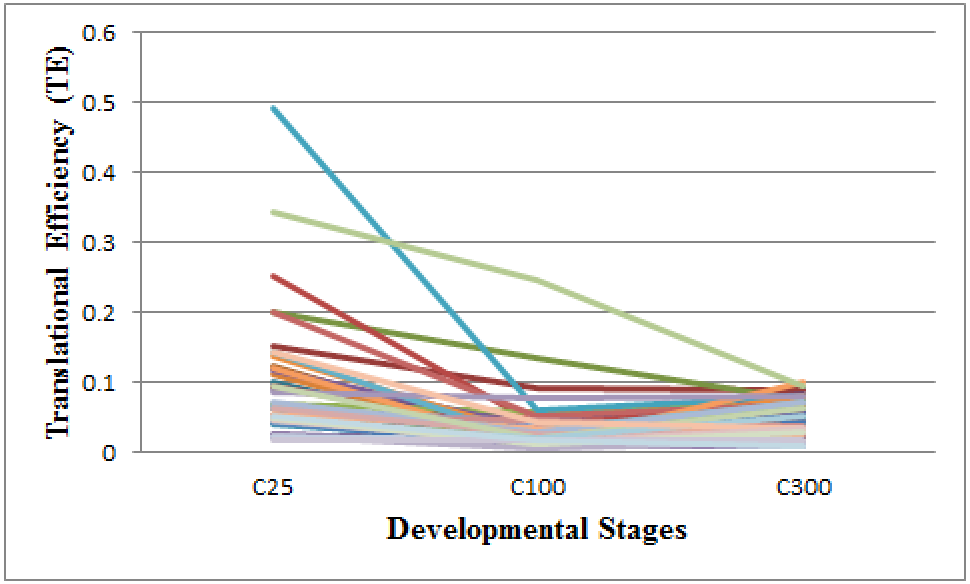

Cluster 2: 47 genes

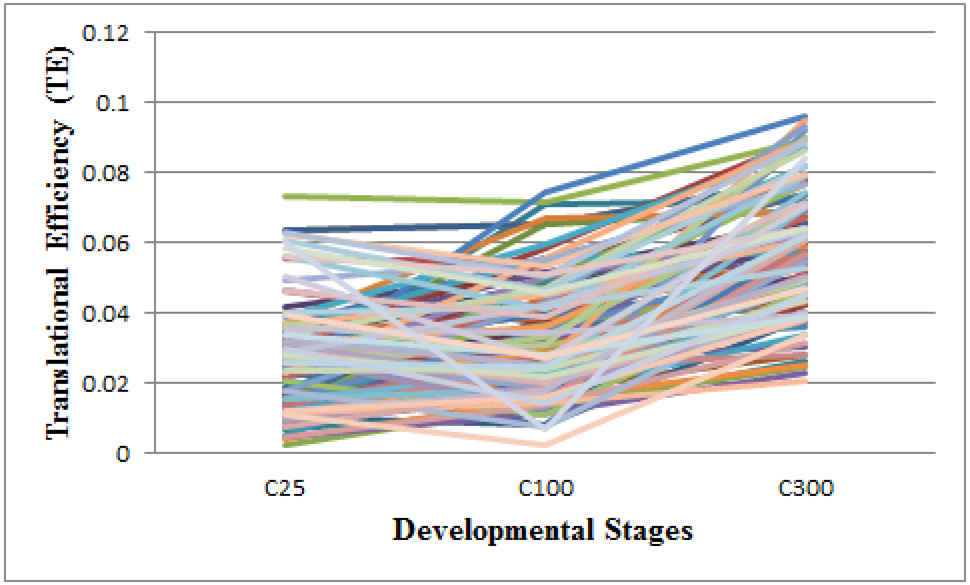

Cluster 3: 121 genes

C25_Cluster 3: 114 genes

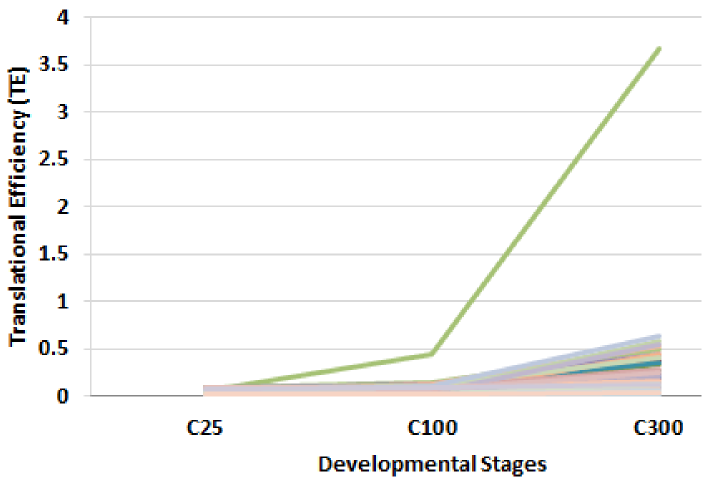

C100_Cluster 3: 110 genes

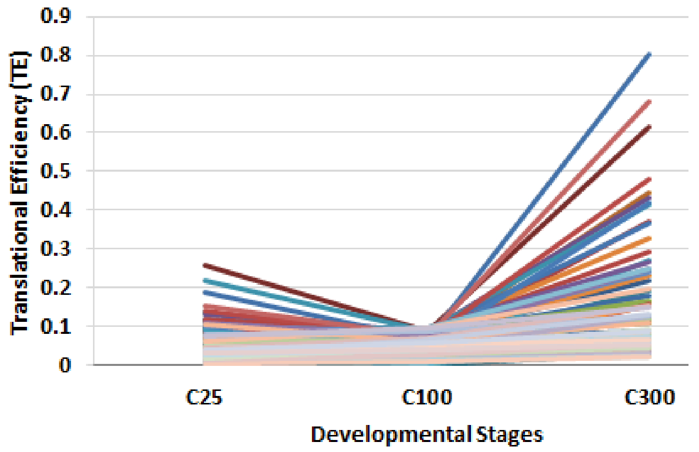

C300_Cluster 3: 121 genes

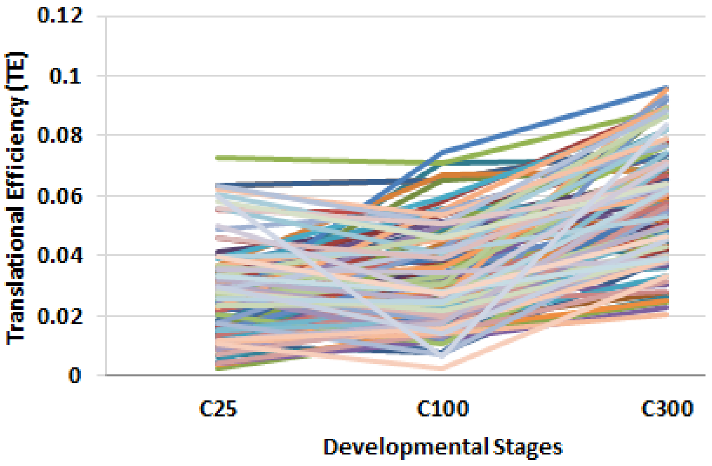

Supplement: S5 File — (PDF) [file pone.0194596.s010.pdf]
